# Eupatilin Promotes Cell Death by Calcium Influx through ER-Mitochondria Axis with SERPINB11 Inhibition in Epithelial Ovarian Cancer

**DOI:** 10.3390/cancers12061459

**Published:** 2020-06-03

**Authors:** Jin-Young Lee, Hyocheol Bae, Changwon Yang, Sunwoo Park, Byung-Soo Youn, Han-Soo Kim, Gwonhwa Song, Whasun Lim

**Affiliations:** 1 Department of Pharmacology and Toxicology, Medical College of Wisconsin, Milwaukee, WI 53226, USA; jylee@mcw.edu; 2 Department of Biotechnology, Institute of Animal Molecular Biotechnology, College of Life Sciences and Biotechnology, Korea University, Seoul 02841, Korea; bhc7@korea.ac.kr (H.B.); ycw117@korea.ac.kr (C.Y.); sunwoojump@korea.ac.kr (S.P.); 3 Osteoneurogen, Inc., Seoul 08501, Korea; byung4jc@gmail.com; 4 Department of Biomedical Sciences, Catholic Kwandong University, Gangneung-si, Gangwon-do 25601, Korea; hansk@cku.ac.kr; 5 Department of Food and Nutrition, Kookmin University, Seoul 02707, Korea

**Keywords:** SERPINB11, ovarian cancer, eupatilin, calcium influx, ER–mitochondria axis

## Abstract

Ovarian cancer is the leading cause of gynecological cancer-related mortality. The anticancer effect of eupatilin, a family of flavonoids, is known in many cancer types, but it is unclear what mechanism it plays in ovarian cancer. In this study, eupatilin promoted cell death of ovarian cancer cells by activating caspases, cell cycle arrest, reactive oxygen species (ROS) generation, calcium influx, disruption of the endoplasmic reticulum (ER)–mitochondria axis with SERPINB11 inhibition, and downregulation of phosphoinositide 3-kinase (PI3K) and mitogen activated protein kinase (MAPK) pathways. Additionally, eupatilin-reduced SERPINB11 expression enhanced the effect of conventional chemotherapeutic agents against ovarian cancer cell progression. Cotreatment with siSERPINB11 and eupatilin increased calcium-ion-dependent apoptotic activity in ovarian cancer cells. Although there were no significant toxic effects of eupatilin on embryos, eupatilin completely inhibited tumorigenesis in a zebrafish xenograft model. In addition, eupatilin suppressed angiogenesis in zebrafish transgenic models. Collectively, downregulating SERPINB11 with eupatilin against cancer progression may improve therapeutic activity.

## 1. Introduction

Ovarian cancer is the leading cause of gynecological cancer-related mortality, with a 10-year survival rate below 30%. In spite of many efforts with numerous drug screenings and therapeutic strategies, the survival rate has not increased over the last three decades [1,2]. The various genetic variations of ovarian cancer and their different sensitivity to chemotherapy make it difficult to formulate treatment strategies for ovarian cancer [3]. Evidence has shown that compounds derived from herbs, fruits, and vegetables can reduce the risk of ovarian cancer and have synergistic effects with chemically synthesized anticancer drugs [4]. Eupatilin is a bioactive flavonoid derived from *Artemisia asiatica*. In recent years, it has been reported that eupatilin has anticancer effects by regulating cell cycle or metastatic capacity in cancers such as gastric cancer, endometrial cancer, and glioma cells [5,6,7]. In addition, eupatilin exerts therapeutic effects in renal cell carcinoma by promoting reactive oxygen species (ROS) production and suppressing the protein kinase B (also known as AKT) signaling pathway [8]. Even in esophageal cancer, eupatilin reduces cell proliferation by inhibiting AKT and mitogen activated protein kinase (MAPK) signaling pathways [9]. However, it is still unclear whether eupatilin would affect the proliferation or cell death of ovarian cancer cells.

Serine protease inhibitors (serpins) are classified into the two largest superfamily clades that are subdivided into 36 extracellular molecules, clade A, and intracellular serpins, clade B, in humans [10,11]. Several SERPIN proteins have been reported to be overexpressed in carcinomas and may contribute to tumor progression. Thus, the expression of SERPIN proteins is considered a potential biomarker in squamous cell carcinoma and colorectal cancer [12,13]. Among the members of SERPIN family, the expression of SERPINB11 was found to be high in ovarian endometrial cancer in chickens, but its role in human ovarian cancer cells is not known yet [14].

We first demonstrated the anticancer potential of eupatilin as a candidate therapeutic agent targeting SERPINB11 in ovarian cancer cells. Eupatilin induced calcium-dependent apoptotic cell death associated with disruption of the endoplasmic reticulum (ER) and mitochondrial axis by targeting SERPINB11 in ovarian cancer cell lines. In-depth pharmacological interactions between drugs and calcium, the ER, and mitochondria are well characterized in various disease models, including anticancer therapies. In physiological processes, communication has been demonstrated between the ER and mitochondria, including calcium and ROS-related cell death and mitophagy [15,16]. Collectively, we proposed that SERPINB11 deficiency with eupatilin enhances therapeutic applications for epithelial ovarian cancer.

## 2. Results

### 2.1. Eupatilin Induces Apoptosis in Ovarian Cancer (OC) Cell Lines

**Figure 1:**
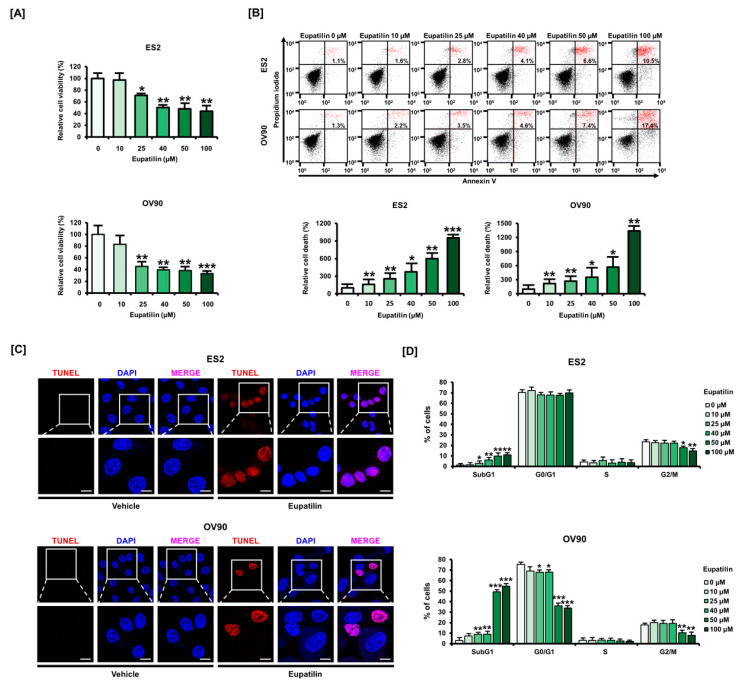
Anticancer effects of eupatilin on ovarian cancer. (**A**) The effects of eupatilin on the cell viability of ES2 and OV90 ovarian cancer cell lines. (**B**) Evaluation of cell death mechanisms in eupatilin-treated ES2 and OV90 cells using annexin-V-fluorescein isothiocyanate (FITC)/propidium iodide (PI) dual-staining analysis. (**C**) Apoptotic cells were stained with terminal deoxynucleotidyl transferase dUTP nick end labeling (TUNEL), a red fluorescent dye. DAPI, a blue fluorescent dye, was used as a positive control in both ES2 and OV90 cells treated with eupatilin. The scale bar indicates 20 μm. (**D**) ES2 and OV90 cells were treated with increasing concentrations of eupatilin and stained with PI to estimate cell cycle distribution using a flow cytometer. The experiments were performed in triplicate. Data represent the mean ± standard deviation, and asterisks indicate that the effect of treatment was statistically significant (* *p* < 0.05, ** *p* < 0.01, and *** *p* < 0.001).

We evaluated the anticancer effect of eupatilin on ovarian cancer cell lines. Our results showed that the cell viability of ES2 and OV90 cells was significantly inhibited by 50 µM eupatilin (Figure 1A). We next assessed annexin/propidium iodide (PI) staining to analyze the features of eupatilin-induced cell death, and our results revealed that PI- and annexin-positive apoptotic populations were increased 2.5- and 1.6-fold by eupatilin treatment in ES2 and OV90 cells, respectively (Figure 1B). Similarly, terminal deoxynucleotidyl transferase dUTP nick end labeling (TUNEL)-positive cells showed that eupatilin induced DNA fragmentation, indicating that eupatilin-mediated cytotoxicity was based on apoptotic cell death in both cell lines (Figure 1C). According to the apoptotic capacity of eupatilin, the cell cycle assay indicated a significant increase in sub-G1 phases with a concomitant reduction in G1 phases (Figure 1D). Taken together, eupatilin induced the cell death of ES2 and OV90 cell lines.

### 2.2. Effects of Eupatilin on ER Stress and Oxidative Stress on Ovarian Cancer Cells

**Figure 2:**
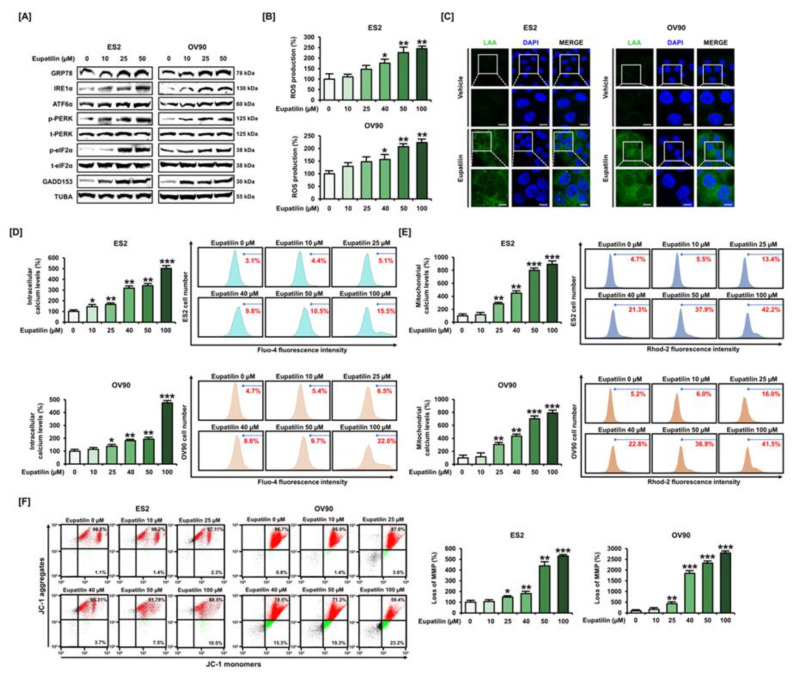
Effects of eupatilin on various aspects of cellular stress in ovarian cancer. (**A**) Western blot of endoplasmic reticulum (ER) stress regulatory proteins after ES2 and OV90 cells were treated with different concentrations of eupatilin. (**B**) The effects of eupatilin on reactive oxygen species (ROS) generation in ES2 and OV90 cells were evaluated by flow cytometry with dichlorofluorescin (DCF) fluorescence signals. (**C**) The effect of eupatilin on lipid peroxidation was determined by immunocytochemistry of linoleamide alkyne (LAA) to indicate lipid peroxidation with green fluorescence in the cytosolic fraction in ES2 and OV90 cells. The scale bar indicates 20 μm. (**D**–**E**) Eupatilin-mediated intracellular (**D**) and mitochondrial (**E**) calcium levels were investigated by flow cytometry with Fluo-4 and Rhod-2 fluorescence signals, respectively, after eupatilin treatment in ES2 and OV90 cells. (**F**) The mitochondrial membrane potential (MMP, ΔΨm) was analyzed by the distribution of red and green fluorescence using JC-1 staining after eupatilin treatment in ES2 and OV90 cells. The experiments were performed in triplicate. Data represent the mean ± standard deviation, and asterisks indicate that the effect of treatment was statistically significant (* *p* < 0.05, ** *p* < 0.01, and *** *p* < 0.001). Detailed information about the western blot can be found in Figure S1.

To evaluate the effects of eupatilin on ER stress, we analyzed the levels of ER stress-related proteins in response to eupatilin treatment. Eupatilin increased ER regulatory protein levels overall; therefore, we concluded that ER stress was induced by eupatilin in OC cells (Figure 2A). After eupatilin treatment, we also found 2.4- and 2.2-fold increases in intracellular ROS in ES2 and OV90 cells, respectively; this was in agreement with the onset of drug-induced cellular stress (Figure 2B). Moreover, lipid peroxidation was increased by 50 μM eupatilin compared to the control, which is consistent with previous results indicating that eupatilin increases ROS levels in OC cells (Figure 2C). As oxidative stress was induced by eupatilin, we additionally determined mitochondrial dysfunction by analyzing change in the calcium ion mitochondria membrane potential (Ψm). The intracellular and mitochondria calcium ion levels were increased at the highest concentration of eupatilin in ES2 and OV90 cells, respectively, compared to the control (Figure 2D,E). Moreover, in both cell lines, JC-1 monomers/aggregate ratios were increased by eupatilin in a dose-dependent manner compared to the control (Figure 2F).

### 2.3. Regulation of Ca^2+^ Leading to Cell Death through the ER–Mitochondria Axis

**Figure 3:**
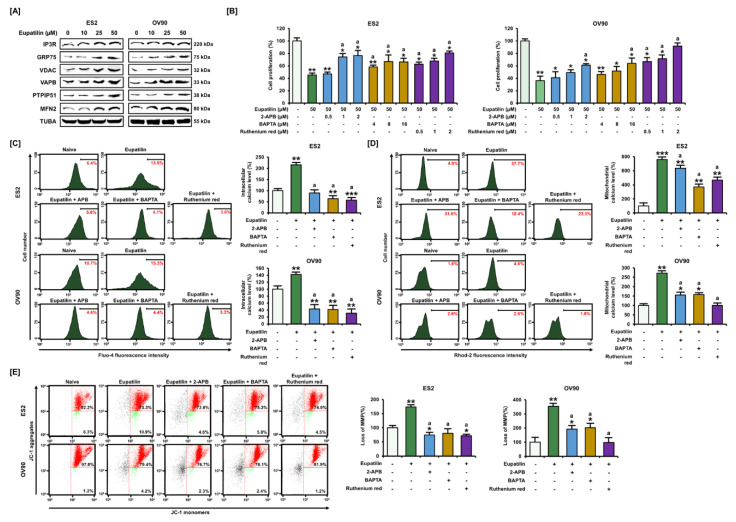
Calcium-induced pathway using specific calcium channel inhibitors with eupatilin treatment. (**A**) Effects of eupatilin on ER–mitochondria axis in ovarian cancer cells by western blot analysis. (**B**) Effects of eupatilin and each calcium channel inhibitor, IP3R (2-APB), intracellular calcium (BAPTA), and sarcoendoplasmic reticulum calcium transport ATPase (SERCA) pump (ruthenium red, RuR) on the proliferation of ES2 and OV90 cells. (**C**,**D**) Effects of eupatilin with each different calcium channel inhibitor on intracellular (**C**) and mitochondrial (**D**) calcium levels in ES2 and OV90 cells. (**E**) Effects of eupatilin with each calcium channel inhibitor on mitochondrial membrane potential (MMP) (ΔΨm) in ES2 and OV90 cells by JC-1 staining. The experiments were performed in triplicate. Data represent the mean ± standard deviation, and asterisks indicate that the effect of treatment was statistically significant (* *p* < 0.05, ** *p* < 0.01, and *** *p* < 0.001). A lowercase letter (a) indicates significant changes (*p* < 0.05) compared with eupatilin alone. Detailed information about the western blot can be found in Figure S2.

As we had demonstrated that eupatilin mediated calcium disruption, we next assessed ER–mitochondria communication by investigating ER–mitochondria tethering proteins. As illustrated in Figure 3A, calcium-releasing complex IP3R-GRP75-VDAC was activated in ES2 and OV90 cells by eupatilin. In addition, the expression of other ER–mitochondria tethering proteins such as VAPB-PTPIP51 and MFN2 increased in eupatilin-treated ovarian cancer cells. To determine cell proliferation by regulating calcium ions, 2-aminoethoxydiphenyl borate (2-ABP), 1,2-bis(o-aminophenoxy) ethane-N,N,N′,N′-tetraacetic acid (BAPTA), and ruthenium red (RuR) were used to target IP3R, intracellular calcium and mitochondrial calcium uniporter (MCU), respectively. Our results showed that the proliferation of OC cells reduced by eupatilin was significantly recovered by pretreatment with 2-ABP, BAPTA, and RuR, implying that eupatilin may induce calcium-dependent apoptosis through IP3R and MCU in OC cells (Figure 3B). Moreover, eupatilin-induced calcium overload was abrogated by pretreatment with 2-ABP, BAPTA, and RuR compared to intracellular calcium levels after treatment with eupatilin alone (Figure 3C). Similarly, the eupatilin-induced accumulation of mitochondrial calcium was decreased by pre-incubation with calcium chelators in comparison to eupatilin alone, suggesting that apoptosis induced by eupatilin was completely dependent on both intracellular and mitochondrial redistribution of calcium (Figure 3D). In addition, the activated depolarization of Ψm by eupatilin was restored to basal levels by 2-ABP, BAPTA, and RuR compared to eupatilin alone in ES2 and OV90 cells (Figure 3E).

### 2.4. Anticancer Signaling Pathway by Eupatilin in ES2 and OV90 Cells

**Figure 4:**
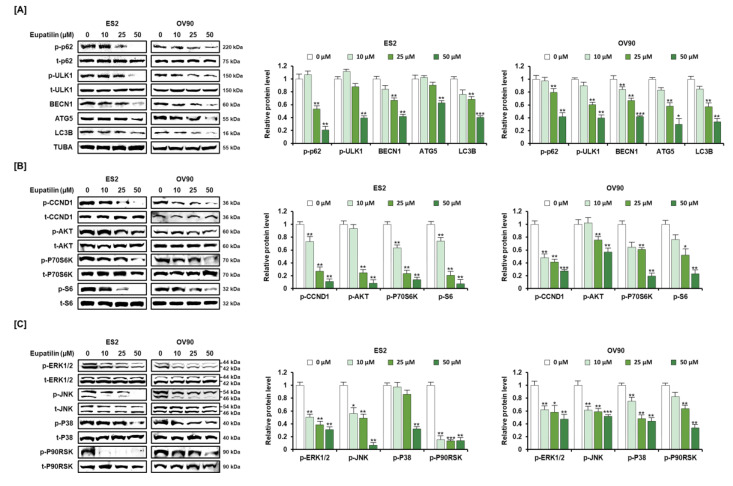
Dose-dependent effects of eupatilin on autophagy, PI3K/AKT, and MAPK signaling in ovarian cancer cells. (**A**) Effects of eupatilin on autophagosome formation in ES2 and OV90 cells. [B-C] Inhibitory effects of eupatilin on PI3K (**B**) and MAPK (**C**) signal transduction in ovarian cancer cells. Each immunoblot was calculated using the normalized ratio of phosphorylated protein relative to the total protein. The experiments were performed in triplicate. Data represent the mean ± standard deviation, and asterisks indicate that the effect of treatment was statistically significant (* *p* < 0.05, ** *p* < 0.01, and *** *p* < 0.001). Detailed information about the western blot can be found in Figure S5.

We then investigated eupatilin-regulated cell signaling involved in cancer progression. In light of ER–mitochondria Ca^2+^ influx through tethering proteins between the ER and mitochondria shown in Figure 3, we next determined autophagy regulatory proteins since autophagy is known to be mainly regulated by ER-mitochondria [17]. Results indicated that tightening ER–mitochondria contacts releasing excessive Ca^2+^ by eupatilin suppressed autophagosome formation in ovarian cancer cells (Figure 4A). Under the same condition, CCND1, PI3K, and MAPK signaling proteins were downregulated in eupatilin-treated ES2 and OV90 cells as compared to non-treated cells (Figure 4B,C). Then, we determined the activities of PI3K and MAPK pathways in response to eupatilin with pharmacological inhibitors such as LY294002 (PI3K inhibitor), U0126 (ERK inhibitor), SP600125 (JNK inhibitor), and SB203580 (P38 inhibitor) (Figures S3 and S4). CCND1 phosphorylation (p-CCND1) was statistically reduced by a combination of eupatilin with LY294002, U0126, or SP600125 compared to eupatilin alone (Figure S3A). The p-AKT was synergistically inhibited by the combination of eupatilin and PI3K inhibitor or ERK1/2 inhibitor in OC cells (Figure S3B). In addition to PI3K and MAPK inhibition, p-P70S6K and p-S6 proteins were mostly blocked in ES2 and OV90 cells (Figure S3C,D). ERK1/2 phosphorylation was synergistically decreased by the combination of eupatilin and U0126 but was highly activated by c-Jun N-terminal kinase (JNK) inhibitor (Figure S3E). JNK expression was completely inhibited by the combination of eupatilin and its inhibitor in OV90 cells, and it was mostly suppressed in ES2 cells (Figure S3F). The p-P38 was completely inhibited by eupatilin alone and by the combined treatment with eupatilin and SB203580 in both ES2 and OV90 cells (Figure S3G). The p-P90RSK was completely inhibited by eupatilin and the combined treatment of eupatilin with U0126 and SB203580 in ES2 cells, and by eupatilin and the combined treatment of eupatilin with LY294002 and U0126 in OV90 cells (Figure S3H). Overall, eupatilin-regulated cascades were shown to be transmitted through crosstalk between PI3K and MAPK, leading to anticancer activities in both cell lines.

### 2.5. Inhibition of SERPINB11 by the Anticancer Effect of Eupatilin, with Apoptosis Induced in OC Cells

**Figure 5:**
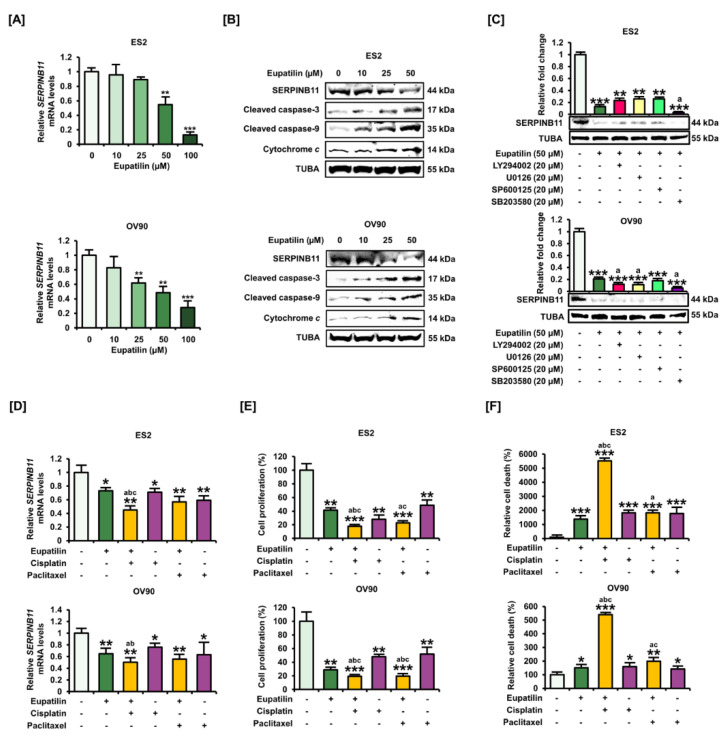
Functional role in SERPINB11 in ovarian cancer treated with eupatilin. (**A**) Dose-dependent effects of eupatilin on *SERPINB11* mRNA expression levels in ovarian cancer cells were determined by quantitative RT-PCR analysis. (**B**) Expression levels of SERPINB11 and apoptosis-related proteins according to eupatilin dosage in ES2 and OV90 cells. (**C**) Expression of SERPINB11 protein was evaluated with each pharmacological inhibitor in eupatilin-treated ovarian cancer cells. (**D**) The mRNA expression of *SERPINB11* according to the separate or combined use of eupatilin, cisplatin, and paclitaxel in ES2 and OV90 cells. (**E**) The synergistic inhibition of cell proliferation in response to the combination of eupatilin with cisplatin or paclitaxel in ES2 and OV90 cells. (**F**) The synergistic induction of cell death in response to the combination of eupatilin with cisplatin or paclitaxel in ES2 and OV90 cells. The experiments were performed in triplicate. Data represent the mean ± standard deviation, and asterisks indicate that the effect of treatment was statistically significant (* *p* < 0.05, ** *p* < 0.01, and *** *p* < 0.001). Lowercase letters indicate statistically significant (*p* < 0.05) effects of treatments: a, compared with eupatilin alone; b, compared with cisplatin alone; and c, compared with paclitaxel alone. Detailed information about the western blot can be found in Figure S6.

We then evaluated the changes in the transcriptional levels of *SERPINB11* in response to eupatilin treatment. Our results showed that transcriptional *SERPINB11* levels in ES2 and OV90 cells were decreased by eupatilin treatment (Figure 5A). We also confirmed the reduction of SERPINB11 and the concomitant induction of cleaved caspase-3, -9, and cytochrome *c*, revealing the mechanism by which eupatilin triggered apoptosis in OC cell lines (Figure 5B). Then, we determined the expression of SERPINB11 in response to eupatilin with pharmacological inhibitors. Eupatilin-reduced SERPINB11 was completely blocked by SB203580 in both ovarian cancer cells (Figure 5C). After identifying systematic apoptosis by eupatilin, we then evaluated synergistic effects between eupatilin and cisplatin or paclitaxel, which are used clinically in OC treatment. The results showed that the transcriptional activation of SERPINB11 was synergistically inhibited by the combination of eupatilin and cisplatin in both OC cells (Figure 5D). Consistent with transcriptional repression of SERPINB11 by combination treatment, synergistic anticancer effects such as reduced cell proliferation and induced cell death were improved by the combination of eupatilin and cisplatin or paclitaxel compared to eupatilin and each therapeutic agent alone in both cell lines (Figure 5E,F).

### 2.6. Effect of siSERPINB11 on Anticancer Activity within OC Cells

**Figure 6:**
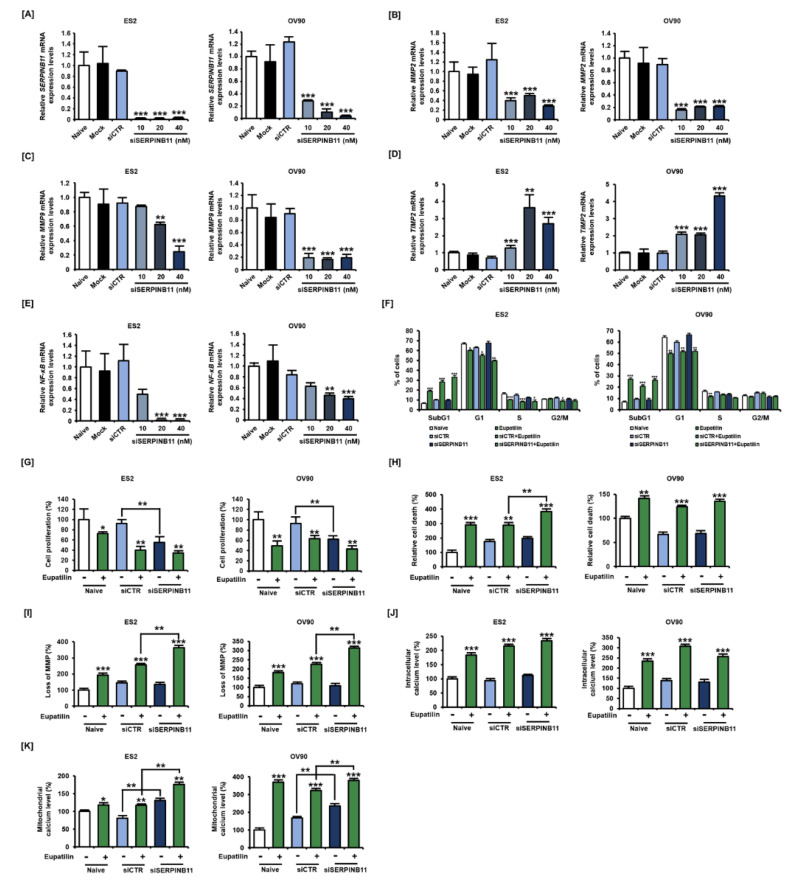
The functional role of SERPINB11 knockdown in the transcriptional levels of ovarian cancer-associated genes and anticancer activity by eupatilin in ovarian cancer cells. (**A**) Dose-dependent inhibition of SERPINB11 using specific target siRNA by transfection using Lipofectamine 2000. ES2 and OV90 cells were transfected with transfection reagent alone (Mock), non-specific control siRNA (siCTR), and naïve cells were used as negative controls. (**B**–**E**) In the absence of SERPINB11 via siSERPINB11 transfection, transcriptional levels of ovarian-cancer-associated genes *MMP2* [B], *MMP9* (**C**), *TIMP2* (**D**), and *NF-κB* (**E**) were evaluated in ES2 and OV90 cells. (**F**) Ovarian cancer cells were treated with an optimal concentration of eupatilin (50 μM) under the presence or absence of SERPINB11, and PI-dependent DNA content was analyzed by flow cytometer to estimate cell cycle distribution. (**G**–**H**) Anti-proliferative effects (**G**) and apoptosis induced (**H**) by eupatilin under SERPINB11 abrogation in ES2 and OV90 cells. (**I**) After eupatilin treatment, the loss of MMP was analyzed by the ratio of red and green fluorescence by JC-1 staining under the presence or absence of SERPINB11. (**J**–**K**) Intracellular (**J**) and mitochondrial calcium (**K**) levels were measured by Fluo-4 and Rhod-2 fluorescence intensity, respectively, under the presence or absence of SERPINB11 in ES2 and OV90 cells. The experiments were performed in triplicate. Data represent the mean ± standard deviation, and asterisks indicate that the effect of treatment was statistically significant (* *p* < 0.05, ** *p* < 0.01, and *** *p* < 0.001).

To validate SERPINB11 as an appropriate anticancer target, we next analyzed the effect of SERPINB11 knockdown on the expression of genes known to promote ovarian cancer. Our results revealed that SERPINB11 transcription was completely abrogated by 10 nM siSERPINB11 in both ES2 and OV90 cells, indicating that the siRNA was effective at inhibiting transcription (Figure 6A). *MMP2* and *MMP9* mRNA levels were reduced by *SERPINB11* inhibition in both cells (Figure 6B,C), whereas *TIMP2*, which is a negative regulator of *MMP2*, was increased by siSERPINB11 (Figure 6D). Additionally, *NF-κB* was gradually reduced in ES2 and OV90 cells by siSERPINB11 (Figure 6E). We then investigated the functional roles of SERPINB11 in eupatilin-induced anticancer activities. Our data demonstrated that the eupatilin-induced increase in the sub-G1 phase was found to be more sensitive with the absence of SERPINB11 in ES2 and OV90 cells, and the combination of siSERPINB11 and eupatilin significantly reduced the G1 phase in ES2 cells compared to siSERPINB11 alone, revealing that SERPINB11 is a major protein target for triggering anticancer activity in ovarian cancer (Figure 6F). Cell proliferation was synergistically inhibited by siSERPINB11 transfection compared to control siRNA (siCTR) (Figure 6G). Additionally, transfection of siSERPINB11 with eupatilin slightly decreased the proliferation of both ES2 and OV90 cells. Regarding cell death, eupatilin without SERPINB11 more significantly induced cell death compared to that with SERPINB11 (Figure 6H). Also, the loss of MMP induced by eupatilin was significantly elevated in ES2 and OV90 cells without SERPINB11 compared to that with SERPINB11 (Figure 6I). Additionally, intracellular and mitochondrial calcium levels were more increased by eupatilin when SERPINB11 was absent (Figure 6J,K).

### 2.7. Expression of SERPINB11 on 3D Tumor Formation and Effects of Eupatilin in Vivo and in Vitro

**Figure 7:**
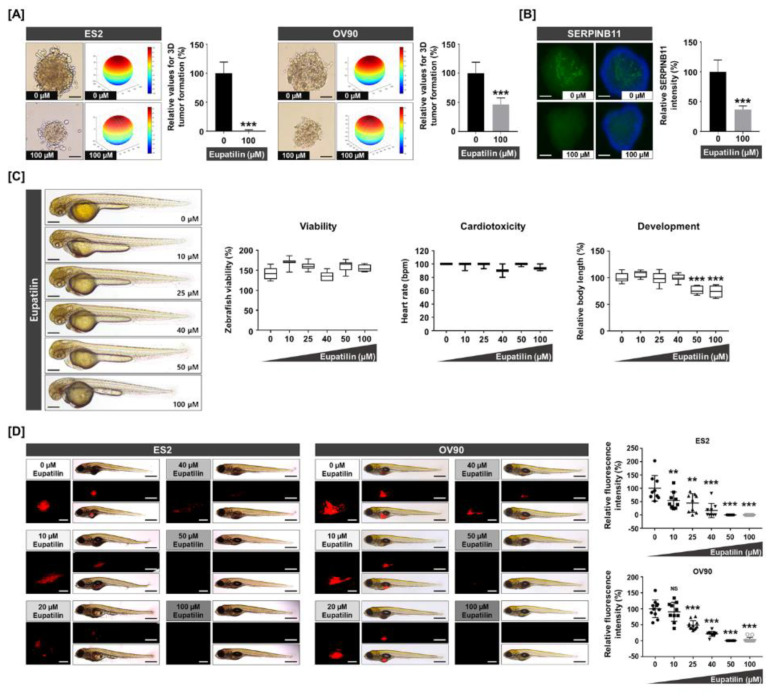
Anticancer effects of eupatilin on in vitro and in vivo 3D anchorage-independent growth and xenograft models. (**A**) The 3D tumor spheroid-forming capacity shows that eupatilin exhibits anticancer potential under anchorage-independent cell growth conditions. The scale bar indicates 20 μm. (**B**) Immunofluorescence of SERPINB11-positive cells (green), which were counterstained with DAPI (blue) on the ES2 tumor spheroid. The scale bar indicates 20 μm. (**C**) Zebrafish embryos of 24 hpf were treated with increasing concentrations of eupatilin for 24 h to analyze morphology, viability, cardiotoxicity, and development as toxicological parameters. The scale bar indicates 250 μm. (**D**) In vivo assay of anticancer effects by eupatilin in a zebrafish xenograft model. Red fluorescence intensities were quantified to determine tumor formation. The scale bar indicates 25 μm in square panels and 100 μm in rectangle panels. The experiments were performed in triplicate. Data represent the mean ± standard deviation, and asterisks indicate that the effect of treatment was statistically significant (** *p* < 0.01 and *** *p* < 0.001).

Based on the previous confirmation obtained in 2D culture, we next assessed the effect of eupatilin in an anchorage-independent culture system to demonstrate its anticancer potential under conditions reflecting a tumor microenvironment. Eupatilin completely prevented spheroid formation in both ES2 and OV90 (Figure 7A). Additionally, SERPINB11 expression was abrogated in ES2 spheroids following eupatilin treatment, in agreement with the results obtained with the 2D culture system (Figure 7B). Since our data suggested that eupatilin had strong potential as an anticancer drug, we conducted safety profiling in response to eupatilin treatment in normal zebrafish to verify the safety of the drug. It did not trigger acute toxicity, as shown by normal morphology and viability in zebrafish, nor cardiotoxicity, as shown by a normal range of heartbeats per min (Figure 7C). To confirm the anticancer potential of eupatilin under 3D conditions, we used an ovarian cancer xenograft model. We found that eupatilin triggered complete abrogation of tumorigenesis in vivo, whereas DMSO (the vehicle used for eupatilin) was unable to reduce the tumor volume in a zebrafish xenograft model with ovarian cancer cells (Figure 7D).

**Figure 8:**
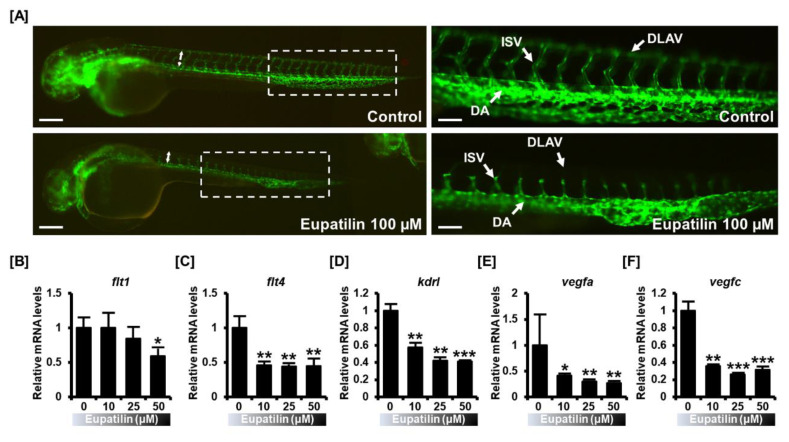
In vivo validation of an inhibitory effect on angiogenesis by eupatilin. (**A**) A zebrafish transgenic model (*fli1:EGFP*) was used to determine the physiological anti-angiogenic effects of eupatilin. Eupatilin-treated embryos indicated dorsal longitudinal anastomotic vessel (DLAV) and dorsal aorta (DA) defects and branching intersegmental vessels (ISVs). The scale bar indicates 300 μm in the first vertical panels and 60 μm in the second vertical panels [B–F] Expression of angiogenesis-associated genes, including *flt1* (**B**), *flt4* (**C**), *kdrl* (**D**), *vegfa* (**E**), and *vegfc* (**F**), were analyzed in eupatilin-treated or untreated zebrafish fli1 embryos. The experiments were performed in triplicate. Data represent the mean ± standard deviation, and asterisks indicate that the effect of treatment was statistically significant (* *p* < 0.05, ** *p* < 0.01, and *** *p* < 0.001).

Next, to examine the contribution of eupatilin to physiological angiogenesis, we analyzed vascular formation in transgenic zebrafish (*fli1:EGFP*) in response to eupatilin. Vascular development was completely interrupted in the presence of eupatilin, whereas the vasculature was well organized in normal conditions (Figure 8A). Interestingly, the dorsal longitudinal anastomotic vessel (DLAV), intersegmental vessel (ISV), and dorsal aorta (DA) regions were completely disrupted by eupatilin treatment. Consistent with these results, the angiogenesis-related genes *flt1*, *flt4*, *kdrl*, *vegfa*, and *vegfc* were significantly inhibited by eupatilin treatment, indicating that eupatilin harbors anti-angiogenetic functions (Figure 8B–F).

## 3. Discussion

In chemotherapy, an integrated approach using various drug combinations is essential to overcome resistances to specific drugs through drug-mediated genetic diversity, and this approach best inhibits the spread of metastasis beyond the original onset of cancer. Cisplatin and paclitaxel, based on taxane and platinum analogues, are the most common chemotherapeutic agents for ovarian cancer. Here, we showed that a combination regimen with eupatilin and cisplatin or paclitaxel was effective. Synergistic inhibition of SERPINB11 expression was observed in response to the combination of eupatilin and the platinum-based anticancer drug, which significantly increased the population of dead cells compared to therapy with eupatilin or cisplatin alone in OV90 and ES2. The combination of eupatilin and the taxane-based anticancer drug significantly reduced SERPINB11 and cell growth in OV90 and ES2; overall, the results indicated that the combination with platinum was more effective than with taxane. Moreover, eupatilin suppressed PI3K and MAPK signal transduction, which are closely involved in cell proliferation and survival in ovarian cancer [18,19]. Blockage of AKT, ERK1/2, JNK and P38 signaling with eupatilin additionally decreased the expression of SERPINB11 protein. Considering that drug repositioning has already been attempted in some papers on ovarian cancer, our data supports drug rearrangement based on the anticancer potential of eupatilin and may provide useful information to accelerate the process for traditional drug development [20].

Eupatilin has been commonly used as a treatment for gastritis and peptic ulcers; mechanistically, eupatilin stabilizes the membrane potential and reduces gastrointestinal motility by affecting the contraction of the gastric muscle [21]. Aside from the main effects exhibited by eupatilin in peptic ulcer disease, several studies have emphasized that eupatilin may have anticancer potential against gastric, renal, and endometrial cancer cells. Gastric cancer growth was hindered by the inhibition of STAT3-triggered VEGF, and tumor invasion was also suppressed by caspase activation and pro-inflammatory cytokine-triggered MMP regulation by eupatilin treatment [6,22]. Eupatilin also activates the apoptotic pathway mediated by the cleavage of caspase-3 in gastric cancer cells, similar to the results verified in ovarian cancer cells in this study [23]. Moreover, cell cycle arrest is one of the routes of eupatilin-induced anticancer effects in gastric cancer and glioma, as demonstrated in ovarian cancer cells [24,25]. Eupatilin also triggered apoptosis by elevating p53 and p21 in agreement with the Bax, Bcl-2, and Parp regulations in human gastric cancer (AGS) cells [23]. Additionally, eupatilin increased ROS generation and apoptosis by abrogating MAPK and AKT in renal cancer [8] and increased mitotic cell cycle arrest by inhibiting the AKT and ERK pathways in endometrial cancer cells [7]. Besides, not only does eupatilin induce canonical apoptosis in cancer cells, but it also attenuates cytokine-induced apoptosis through autophagy induction to protect chondrocytes [26]. Autophagy is known to be regulated by ER–mitochondrial signaling [17], and our results suggest that the autophagy is also associated with eupatilin-mediated apoptosis and cell signal transduction in ovarian cancer. This is in line with the diverse therapeutic function of eupatilin in various diseases through transinteraction. Another study also suggests that eupatilin targets the AKT signaling pathway in cancer cells, causing cell cycle arrest [27]. Treatment with eupatilin in the xenograft mouse model for esophageal cancer inhibits the activity of AKT and ERK1/2 in tissues [9]. Moreover, a recent study showed that eupatilin can target microRNAs in cancer cells to induce apoptosis, so further studies are needed to determine if eupatilin may have anticancer effects by modulating microRNAs in ovarian cancer cells [28]. Accumulated evidence suggests that eupatilin inhibits cell proliferation and induces apoptosis as a common mechanism in various cancer cells. In this study, it was confirmed that the mechanism of action of eupatilin previously reported in other cancer cells can be applied to ovarian cancer cells, along with the discovery of newly identified physiological mechanisms and target proteins of eupatilin. Even recently, it has been suggested that eupatilin may have therapeutic effects in cancers derived from female reproductive organs, such as cervical cancer [29]. Furthermore, Jurkat T-cell immune suppression was induced by eupatilin-mediated calcium oscillation and NF-κB inhibition, which is a primary aspect of the pharmacological activity of eupatilin [30].

Regarding drug safety, Eupatilin (Stillen^®^, Seoul, South Korea) has already been approved by the Food and Drug Administration (FDA, Silver Spring, MD, USA) as a treatment for gastritis and digestive ulcers, this means that the safety concerns are relatively low. Moreover, our results from in vivo safety assessment with zebrafish embryos support that 50 μM eupatilin might be within safety concentrations. Nevertheless, it is unclear whether these doses are completely stable in humans since no studies have demonstrated the pharmacokinetics of eupatilin within effective anticancer concentrations. However, the results of several studies conducted on human cells suggest that eupatilin is not toxic to normal cells, such as adipocytes [31], unlike cancer cells. Rather, in normal cells such as ileal smooth muscle cells [32] and kidney epithelial cells [33], eupatilin is reported to have a protective effect. Therefore, at this dose, eupatilin is presumed to be safe in normal human tissues, although further studies are required.

The oncogenic functions of SERPIN proteins have already been studied in several articles. Among the members of the serpin family, SERPINA1 was suggested as a biomarker for gastric and cutaneous squamous cell carcinoma harboring tumor invasion and migration with poor prognosis [12]. SERPINB3, -4, -6, and -13 were discovered as cathepsin inhibitors to promote tumor formation in squamous cell carcinomas and autoimmune diabetes [34,35,36,37]. In addition, SERPINB5 was demonstrated to be a biomarker for colorectal cancer that interacts with carcinoembryonic antigen (CEA), which has been already approved as a clinical marker [13], and was shown to increase tumor cell invasion and angiogenesis in human mammary epithelial cells [38]. For SERPINB11, overexpression was discovered in the mouse uterus and was found to play a role in female reproductive function in mice [39]. However, there is no clear evidence of the anticancer activities exhibited by the regulation mechanism of SERPINB11 in ovarian cancer.

In the present study, the function of mitochondria was investigated through JC-1 dye. Under hyperpolarization, the increasing magnitude of the internal negative charge membrane potential, JC-1 dye may flow from the medium, decreasing overall fluorescence in the cell suspension because of the dye uptake in the cells. On the other hand, mitochondrial membrane depolarization may release the dye, increasing fluorescence signals. JC-1 can selectively enter into mitochondria and reversibly change the color from green to red as the membrane potential increases because it is a lipophilic, cationic dye. In healthy cells with high MMP (ΔψM), JC-1 spontaneously forms complexes known as J-aggregates with intense red fluorescence [40]. Eupatilin significantly increased loss of MMP in ES2 and OV90 cells. Thus, JC-1 dye flowed from eupatilin uptake cells to the medium, increasing the fluorescence signals precisely detected by flow cytometry. We first demonstrated the anticancer potential of eupatilin, which induced calcium-dependent apoptosis associated with disruption of the ER–mitochondrial axis by targeting SERPINB11 in ovarian cancer cell lines. Xu et al. recently identified anticancer effects via calcium, ER, and MCU by targeting IP3R, GRP75, and MCU in a doxorubicin-treated nephropathy model [15,16]. Although several investigations showed the anticancer potential of eupatilin, eupatilin-mediated ER and MCU axis disruption and its associated functions with SERPIN family proteins had yet to be assessed. Hence, our results first suggested that eupatilin-induced apoptosis by the disruption of calcium homeostasis through ER–mitochondria contact in ovarian cancer cells. Considering that IP3R inhibitor (2-APB), intracellular calcium chelator (BAPTA), and sarcoendoplasmic reticulum calcium transport ATPase (SERCA) pump inhibitor (RuR) abrogated eupatilin-induced apoptosis, the disruption of calcium homeostasis could be one of the major anticancer properties exhibited by eupatilin. Additionally, calcium influx is well known to stimulate ROS production, leading to apoptosis [41]. Because the ER is a major axis inducing calcium release into the cytoplasm and mitochondria through IP3R [42], we further identified the effect of eupatilin on ER stress and SERPINB11-associated calcium levels. We clearly showed that eupatilin increased ER stress, and the abrogation of SERPINB11 by target siRNA did not affect calcium levels intracellularly but stimulated those in mitochondria. These results indicate that SERPINB11 is a key regulator of eupatilin-induced anticancer activity, and the disruption of calcium homeostasis by eupatilin depends on the SERPINB11-associated ER and MCU axis.

In addition, antitumorigenesis and the characteristics of metastasis are extremely important for anticancer strategies against ovarian cancer. The anti-angiogenic function of eupatilin was investigated in human hepatocellular models through MMP2 and VEGF inhibition, but there is still no evidence in in vivo models [43]. Here, we demonstrated the effects of eupatilin on physiological angiogenesis using a transgenic zebrafish model, which contributes to the overall understanding of the anticancer potential of eupatilin. In addition, eupatilin has been shown to reduce tumor formation through a complete reduction of SERPINB11 in adhesion-independent cancer cell growth. This suggests that eupatilin plays a functional role in the prevention of tumorigenesis and angiogenesis within ovarian cancer cells.

## 4. Materials and Methods

### 4.1. Cell Culture

OV90 (serous adenocarcinoma; catalog number: CRL-11732) and ES2 (clear cell carcinoma; catalog number: CRL-1978) ovarian cancer cells were purchased from the ATCC (American Type Culture Collection) (Manassas, VA, USA). Cells were maintained in McCoy’s 5A Medium (catalog number: 16600-082, Gibco, Waltham, MA, USA) containing 10% fetal bovine serum (FBS) and 1% penicillin-streptomycin at 37 °C in a CO_2_ incubator. For the experiments, monolayer cultures of OV90 and ES2 cells were grown in the culture medium to reach about 70% confluency in 100 mm tissue culture dishes.

### 4.2. Reagents

Chemically synthesized eupatilin was obtained from Syngene International Ltd. (Bangalore, India), dissolved in DMSO, and stored in aliquots at −20 °C. Cisplatin, paclitaxel, and 2-APB were purchased from Sigma (St. Louis, MO, USA). Antibodies used in the present study are listed in Table 1. Inhibitors for ERK1/2 (U0126), JNK (SP600125), and P38 (SB203580) were obtained from Enzo Life Sciences (Farmingdale, NY, USA), and PI3K/AKT inhibitor (LY294002) was obtained from Cell Signaling Technology (Danvers, MA, USA). BAPTA was purchased from Santa Cruz Biotechnology (Santa Cruz, CA, USA), and RuR was purchased from Abcam (Cambridge, UK).

### 4.3. Proliferation Assay

Proliferation assays were conducted in ES2 and OV90 cells in response to different treatments using a Cell Proliferation ELISA, BrdU kit (Cat No: 11647229001, Roche, Basel, Switzerland) according to the manufacturer’s protocol. Briefly, ES2 and OV90 cells were seeded in a 96-well plate and then incubated for 24 h in serum-free medium. Cells were then treated with different concentrations of eupatilin with or without signaling inhibitors in a final volume of 100 μL/well. After 48 h of incubation, 10 μM bromo-2’-deoxyuridine (BrdU) was added to the cell culture and the cells were incubated for an additional 2 h at 37 °C. After labeling of cells with BrdU, the fixed cells were incubated with an anti-BrdU-peroxidase (POD) working solution for 90 min. The anti-BrdU-POD binds to BrdU incorporated in newly synthesized cellular DNA, and these immune complexes were detected by their reaction with the 3,3’,5,5’-tetramethylbenzidine (TMB) substrate. The absorbance values of the reaction product were determined by measuring the absorbance at 370 nm and 492 nm using an ELISA reader.

### 4.4. Determination of Apoptosis by Annexin V and Propidium Iodide (PI) Staining

Induction of apoptosis in OV90 and ES2 cells by eupatilin (0, 10, 25, 40, 50, and 100 μM) and knockdown of SERPINB11 with or without eupatilin (50 μM) was analyzed using the Fluorescein isothiocyanate (FITC) Annexin V Apoptosis Detection Kit I (BD Biosciences, Franklin Lakes, NJ, USA). After treatment for 48 h at 37 °C in a CO_2_ incubator was applied to the cells (5 × 10^5^ cells) seeded on 6-well plates, supernatants were removed from culture dishes and adherent cells detached with trypsin-EDTA. The cells were collected by centrifugation, washed with PBS, and resuspended using 1× binding buffer at 1 × 10^6^ cells/mL. Then, 100 μL of the cell suspension (1 × 10^6^ cells) was transferred to a 5 mL culture tube and incubated with 5 μL FITC Annexin V and 5 μL PI for 15 min at room temperature in the dark. Then, 400 μL of 1× binding buffer was added in a 5 mL culture tube. The fluorescence intensity was analyzed using the Guava^®^ easyCyte™ flow cytometer (Merck, Kenilworth, NJ, USA). The data represent three independent experiments.

### 4.5. Terminal Deoxynucleotidyl Transferase dUTP Nick End Labeling (TUNEL) Assay

ES2 and OV90 cells (3 × 10^4^ cells per 300 μL) were prepared on confocal dishes (SPL Life Science, Gyeonggi-do, Korea), and then 50 µM eupatilin was treated for 48 h at 37 °C in a CO_2_ incubator. After treatment, the cells were air-dried and fixed with 4% paraformaldehyde for 1 h at 25 °C. The cells were briefly rinsed with PBS and permeabilized by 0.1% Triton X-100 in 0.1% sodium citrate for 2 min on ice. Then, the cells were stained by tetramethylrhodamine (TMR) red, for 1 h at 37 °C in the dark. Cells were then washed with PBS and overlaid with DAPI. Fluorescence was assessed using an LSM710 (Carl Zeiss, Oberkochen, Germany) confocal microscope fitted with a digital microscope AxioCam camera with Zen2009 software (Carl Zeiss, Oberkochen, Germany).

### 4.6. Cell Cycle Analysis

To examine the distribution of Sub-G1, G1, S, and G2/M phases of eupatilin-treated cells and siSERPINB11-transfected cells with or without eupatilin, we stained OV90 and ES2 cells with propidium iodide (PI; BD Biosciences, Franklin Lakes, NJ, USA) in the presence of 100 μg/mL RNase A (Sigma, St.Louis, MO, USA). Fluorescence was analyzed using the Guava^®^ easyCyte™ flow cytometer (Merck, Darmstadt, Germany).

### 4.7. Western Blot Analysis

Protein concentrations in whole-cell extracts were determined using the Bradford protein assay (Bio-Rad, Hercules, CA, USA) with bovine serum albumin (BSA) as the standard. Proteins were denatured, separated using SDS-PAGE, and transferred to nitrocellulose. Blots were developed by chemiluminescence reaction (SuperSignal West Pico, Pierce, Rockford, IL, USA) and detected by the ChemiDoc EQ system (Bio-Rad, Hercules, CA, USA).

### 4.8. Cellular ROS Determination

Intracellular ROS production was estimated using 2’,7’-dichlorofluorescein diacetate (DCFH-DA, Sigma), which converts to fluorescent 2’,7’-dichlorofluorescein (DCF) in the presence of peroxide. Cells were treated with eupatilin (0, 10, 25, 40, 50, and 100 μM) for 24 h. Cells were then detached using trypsin-EDTA, collected by centrifugation, and washed with phosphate buffered saline (PBS). Furthermore, cells were treated with 10 μM DCFH-DA for 30 min at 37 °C, washed twice with PBS, and the DCF fluorescence intensity was analyzed using the Guava^®^ easyCyte™ flow cytometer (Merck). The data represent three independent experiments.

### 4.9. Lipid Peroxidation

For lipid peroxidation analysis, a Click-iT Lipid Peroxidation Imaging Kit (Invitrogen, Carlsbad, CA, USA) was applied in 50 μM eupatilin-treated ES2 and OV90 cells, and cells were fixed by 3.7% formaldehyde and were permeabilized by 0.5% Triton X-100. Cells were then labeled by Alexa Fluor 488 Azide and briefly washed with PBS and overlaid with DAPI. Fluorescence was assessed using an LSM710 (Carl Zeiss) confocal microscope fitted with a digital microscope AxioCam camera with Zen2009 software.

### 4.10. Measurement of Intracellular Free Ca^2+^ Concentration

ES2 and OV90 cells (5 × 10^5^ cells) were seeded on 6-well plates and incubated for 24 h in serum-free medium until cells reached 70–80% confluency. Then, cells were treated with eupatilin, 2-APB, BAPTA, and ruthenium red for 48 h at 37 °C in a CO_2_ incubator. Supernatants were removed from culture dishes, and adherent cells were detached with trypsin-EDTA. The cells were collected by centrifugation. Collected cells were resuspended in 3 μM Fluo-4 (Invitrogen) and incubated at 37 °C in a CO_2_ incubator for 20 min. The stained cells were washed with PBS. Fluorescence intensity was analyzed using the Guava^®^ easyCyte™ flow cytometer (Merck).

### 4.11. Determination of Mitochondrial Free Ca^2+^ Levels

ES2 and OV90 cells (5 × 10^5^ cells) were seeded in 6-well plates and incubated for 24 h in serum-free medium until they reached 70–80% confluency. Then, cells were treated with eupatilin, 2-APB, BAPTA, and ruthenium red for 48 h at 37 °C in a CO_2_ incubator. Supernatants were removed from culture dishes, and adherent cells were detached with trypsin-EDTA. The cells were collected by centrifugation. Collected cells were resuspended in 3 μM Rhod-2 AM (Invitrogen) diluted with Hank’s Balanced Salt Solution (HBSS, Gibco) and incubated at 4 °C for 30 min. The stained cells were washed with HBSS. Fluorescence intensity was analyzed using the Guava^®^ easyCyte™ flow cytometer (Merck).

### 4.12. JC-1 Mitochondrial Membrane Potential Assay

The JC-1 mitochondrial membrane potential (MMP) was analyzed using a mitochondria staining kit (Sigma). The cells were treated with eupatilin, 2-APB, BAPTA, and ruthenium red for 48 h at 37 °C in a CO_2_ incubator. Supernatants were removed from the culture dishes, and adherent cells were detached with trypsin-EDTA. Cells were collected by centrifugation and resuspended in a staining solution containing 200× JC-1 and 1× staining buffer. The suspension was incubated at 37 °C in a CO_2_ incubator for 20 min. The stained cells were collected by centrifugation, washed once with 1× JC-1 staining buffer, centrifuged again, and then resuspended in 1 mL staining buffer. Fluorescence intensity was analyzed using the Guava^®^ easyCyte™ flow cytometer (Merck).

### 4.13. RNA Extraction and Quantitative RT-PCR

Total cellular RNA was isolated using Trizol reagent (Invitrogen, Waltham, MA, USA) according to the manufacturer’s instructions. Complementary DNA (cDNA) was synthesized using total RNA (1 µg) and AccuPower^®^ RT PreMix (Bioneer, Daejeon, Korea). Gene expression levels were measured using SYBR^®^ Green (Sigma, St. Louis, MO, USA) and the StepOnePlus™ Real-Time PCR System (Applied Biosystems, Foster City, CA, USA) as described in previous study [14]. Primers used for cDNA synthesis are listed in Table 2.

### 4.14. Small Interference RNA (siRNA) Transfection

*SERPINB11* siRNA was purchased from Bioneer (Daejeon, Korea), and nontargeting control siRNA was purchased from Origene (Rockville, MD, USA). Cells were seeded at 1 × 10^5^ cells/well in 6-well plates and incubated until they reached 70–80% confluency. The cells were transfected with siRNA (control siRNA, final concentration 10 nM; siRNA-*SERPINB11*, final concentration 40 nM) using Lipofectamine 2000 (Invitrogen) according to the manufacturer’s instructions. In brief, siRNA and lipofectamine were diluted to 1 mL total in Opti-MEM (Gibco-BRL Life Technologies, Waltham, MA, USA) per well. After pre-incubation for 5 min at room temperature, both solutions were mixed and incubated for an additional 20 min. The ES2 and OV90 cells were treated and incubated for 24 h, and the medium was then replaced with new medium with or without 50 μM eupatilin.

### 4.15. Spheroid Formation

Next, 10,000 ES2 or OV90 cells were seeded in 100 µL complete medium at indicated concentrations of eupatilin in 96-well microtiter μ-bottom plates. ES2 and OV90 cells were incubated for five days at 37 °C and 5% CO_2_ until spheroids formed.

### 4.16. Immunofluorescence Analysis

The effects of eupatilin on SERPINB11 expression in ES2 and OV90 spheroids were probed with goat anti-human polyclonal SERPINB11 at a final dilution of 1:100 (2 µg/mL). They were then incubated with rabbit anti-goat IgG Alexa 488 (Invitrogen, Carlsbad, CA, USA) at a 1:200 dilution for 1 h at room temperature. Spheroids were then washed using 0.1% BSA in PBS and overlaid with DAPI. For each primary antibody, images were captured using a LSM710 (Carl Zeiss) confocal microscope. The intensity staining was assessed using Metamorph Offline software (Molecular Devices, San Jose, CA, USA).

### 4.17. In Vivo Toxicity and Xenograft Analysis

Wild-type *Danio rerio* were maintained according to Korea University guidelines at 28.5 °C with 10 h/14 h dark/light cycles. To evaluate the toxicity of eupatilin, zebrafish embryos were obtained from natural spawning between one male and two female adult fish. Ten embryos per well were transferred to 24-well plates under each condition and cultured for 24 h in 0.3% Danieau’s buffer (1740 mM NaCl, 21 mM KCl, 12 mM MgSO_4_·7 H_2_O, 18 mM Ca(NO_3_)_2_), and 150 mM HEPES), then eupatilin-exposed zebrafish larvae were evaluated for an additional 48 h. Viability, heartbeat, and malformation were evaluated as toxicity indicators.

### 4.18. Angiogenesis Analysis of Blood Vessel Development in Transgenic Zebrafish

Transgenic Fli: larvae were treated with the indicated concentration of eupatilin for 48 h, and then green fluorescent protein (GFP) expression patterns of ten of zebrafish larvae per condition were evaluated. Pictures were obtained using a fluorescence microscope.

### 4.19. Statistics

Statistical analyses were performed using SPSS software version 19.0 (SPSS Inc., Chicago, IL, USA), and a *p*-value < 0.05 was defined as statistically significant.

## 5. Conclusions

In conclusion, we elucidated the anticancer activities exhibited by eupatilin, which induced calcium-dependent apoptotic cell death associated with disruption of the ER and mitochondrial axis by targeting SERPINB11 in ovarian cancer cells. Moreover, we demonstrated the anticancer ability of eupatilin on anchorage-independent cancer cell growth using in vitro and in vivo models. Altogether, our results provide the first pre-clinical evidence for the anticancer potential of eupatilin by targeting SERPINB11 in ovarian cancer.

## Figures and Tables

**Table 1:** List of primary antibodies used in the western blot analyses.

Primary Antibodies	Dilution	Supplier	Catalog Number
Phospho-AKT (SER^473^)	1:1000	Cell Signaling	4060
AKT	1:1000	Cell Signaling	9272
Phospho-ERK1/2 (Thr^202^/Tyr^204^)	1:1000	Cell Signaling	9101
ERK1/2	1:1000	Cell Signaling	4695
Phospho-JNK (Thr^183^/Tyr^185^)	1:1000	Cell Signaling	4668
JNK	1:1000	Cell Signaling	9252
Phospho-P38 (Thr^180^/Tyr^182^)	1:1000	Cell Signaling	4511
P38	1:1000	Cell Signaling	9212
Phospho-P70S6K (Thr^421^/Ser^424^)	1:1000	Cell Signaling	9204
P70S6K	1:1000	Cell Signaling	9202
Phospho-S6 (Ser^235/236^)	1:1000	Cell Signaling	2211
S6	1:1000	Cell Signaling	2217
Phospho-Cyclin D1 (Thr^286^)	1:1000	Cell Signaling	3300
Cyclin D1	1:1000	Cell Signaling	2922
Phospho-P90RSK (Thr^573^)	1:1000	Cell Signaling	9346
P90RSK	1:1000	Cell Signaling	9355
IRE1α	1:1000	Cell Signaling	3294
Phospho-eIF2α (Ser^51^)	1:1000	Cell Signaling	3398
eIF2α	1:1000	Cell Signaling	5324
Phospho-PERK (Thr^981^)	1:1000	Santa Cruz	sc-32577
PERK	1:1000	Santa Cruz	sc-13073
ATF6α	1:1000	Santa Cruz	sc-166659
GADD153	1:1000	Santa Cruz	sc-7351
GRP78	1:1000	Santa Cruz	sc-13968
SERPINB11	1:1000	Santa Cruz	sc-85140
Cleaved caspase-3	1:1000	Cell Signaling	9664
Cleaved caspase-9	1:1000	Cell Signaling	9501
Cytochrome *c*	1:1000	Cell Signaling	11940
TUBA	1:2000	Santa Cruz	sc-5286

**Table 2:** Primer information for quantitative RT-PCR.

Gene		Primer Sequence (5′ →β 3′)		Size (bp)
**Human**	*COX2* *(NM_000963.4)*	Forward	TTCTCCTGCCTACTGGAAGC	106
		Reverse	ACAGCCCTTCACGTTATTGC	
	*MMP2* (NM_001127891.2)	Forward	GGCATTCAGGAGCTCTATGG	137
		Reverse	ATCTCACCACGGATCTGAGC	
	*MMP9* (NM_004994.3)	Forward	TTGACAGCGACAAGAAGTGG	145
		Reverse	TCAGTGAAGCGGTACATAGGG	
	*NF-κB* (NM_001165412.1)	Forward	TCTGTGTTTGTCCAGCTTCG	115
		Reverse	GCTTCTGACGTTTCCTCTGC	
	*SERPINB11* (NM_001291278.1)	Forward	CATTCCGAGTTTGGTGTCG	102
		Reverse	AAATGCCATCGTCTTTGTCC	
	*TIMP2* (NM_003255.5)	Forward	AGAAGAGCCTGAACCACAGG	119
		Reverse	CTCTGTGACCCAGTCCATCC	
**Zebrafish**	*flt1*(BC139515.1)	Forward	CTGGTTATTCGGGATGTTGC	121
		Reverse	TTTGGGGCTTCACATTTACC	
	*flt4* (AY833404.1)	Forward	TCACAACTGGATGGATTTGG	100
		Reverse	GCCGACAGTCTTTTCTTTGC	
	*kdrl* (NM_131472.1)	Forward	CCTGATCCACAACTGCTTCC	142
		Reverse	CACACGACTCAATGCGTACC	
	*vegfa* (AF016244.1)	Forward	ATTCATACCCAGCAGCTTCG	137
		Reverse	GCAGACAGATGGAGGAGAGC	
	*vegfc* (AF466147.1)	Forward	GATGTGGGGAAAGAGTTTGG	112
		Reverse	TGATGTTCCTGCACTGAAGC	

## Supplementary Materials

The following are available online at https://www.mdpi.com/2072-6694/12/6/1459/s1. Figure S1: Detailed information about the western blot in Figure 2, Figure S2: Detailed information about the western blot in Figure 3, Figure S3: Dose-dependent effects of pharmacological inhibitors and eupatilin on PI3K/AKT and MAPK signaling in ovarian cancer cells, Figure S4: Detailed information about the western blot in Figure S3, Figure S5: Detailed information about the western blot in Figure 4, Figure S6: Detailed information about the western blot in Figure 5.
