# Supplementary material for: Eupatilin Promotes Cell Death by Calcium Influx through ER-Mitochondria Axis with SERPINB11 Inhibition in Epithelial Ovarian Cancer
Source: Cancers (Basel). 2020 Jun 3;12(6):1459. doi: 10.3390/cancers12061459 (PMC7353024; doi:10.3390/cancers12061459)
Supplement: Supplementary file 1 [file cancers-12-01459-s001.pdf]

Supplementary Materials

Eupatilin Promotes Cell Death by Calcium Influx through ER-Mitochondria Axis with SERPINB11 Inhibition in Epithelial Ovarian Cancer

Jin-Young Lee, Hyocheol Bae, Changwon Yang, Sunwoo Park, Byung-Soo Youn, Han-Soo Kim, Gwonhwa Song and Whasun Lim

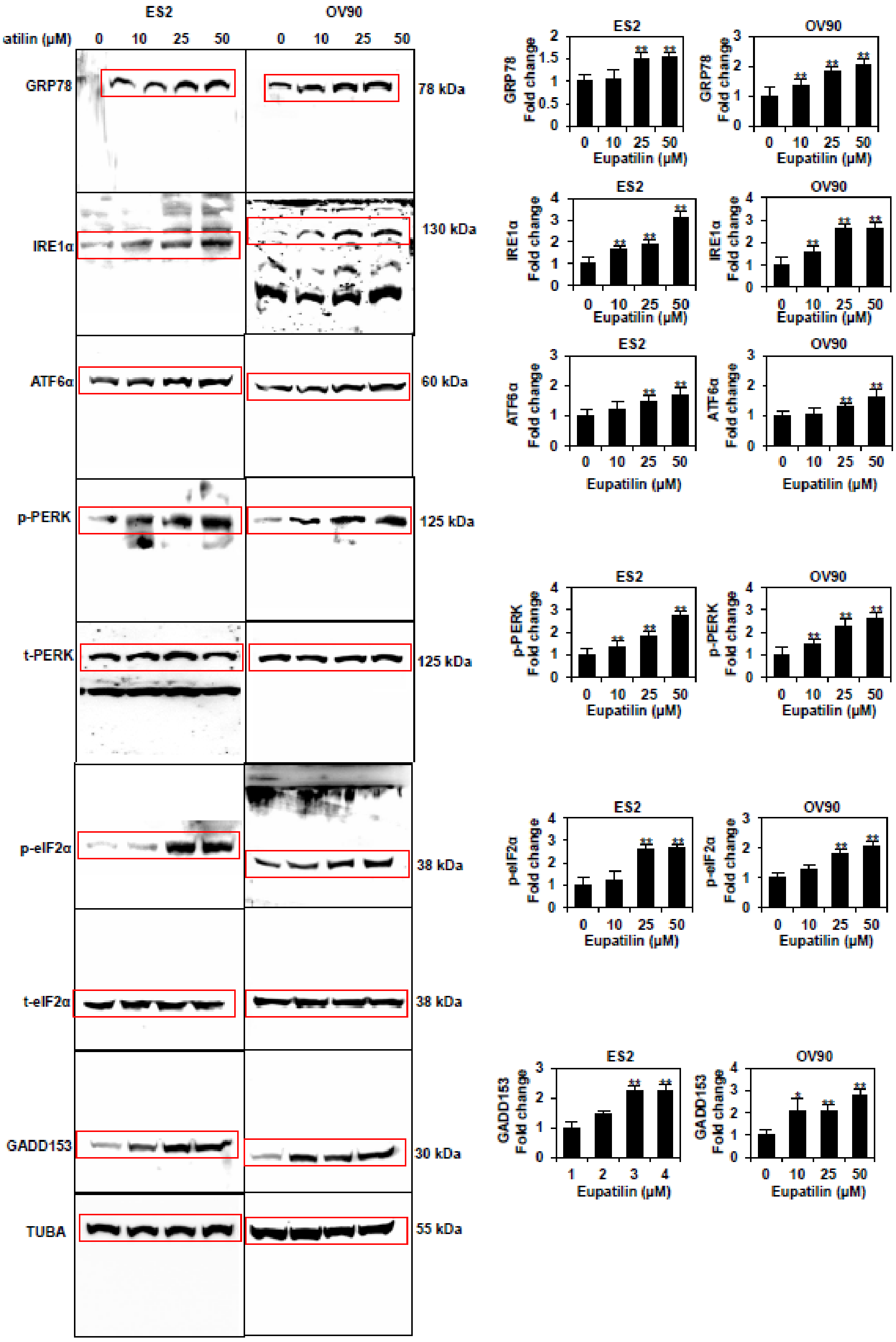

Figure S1. Detailed information about western blot in Figure 2.

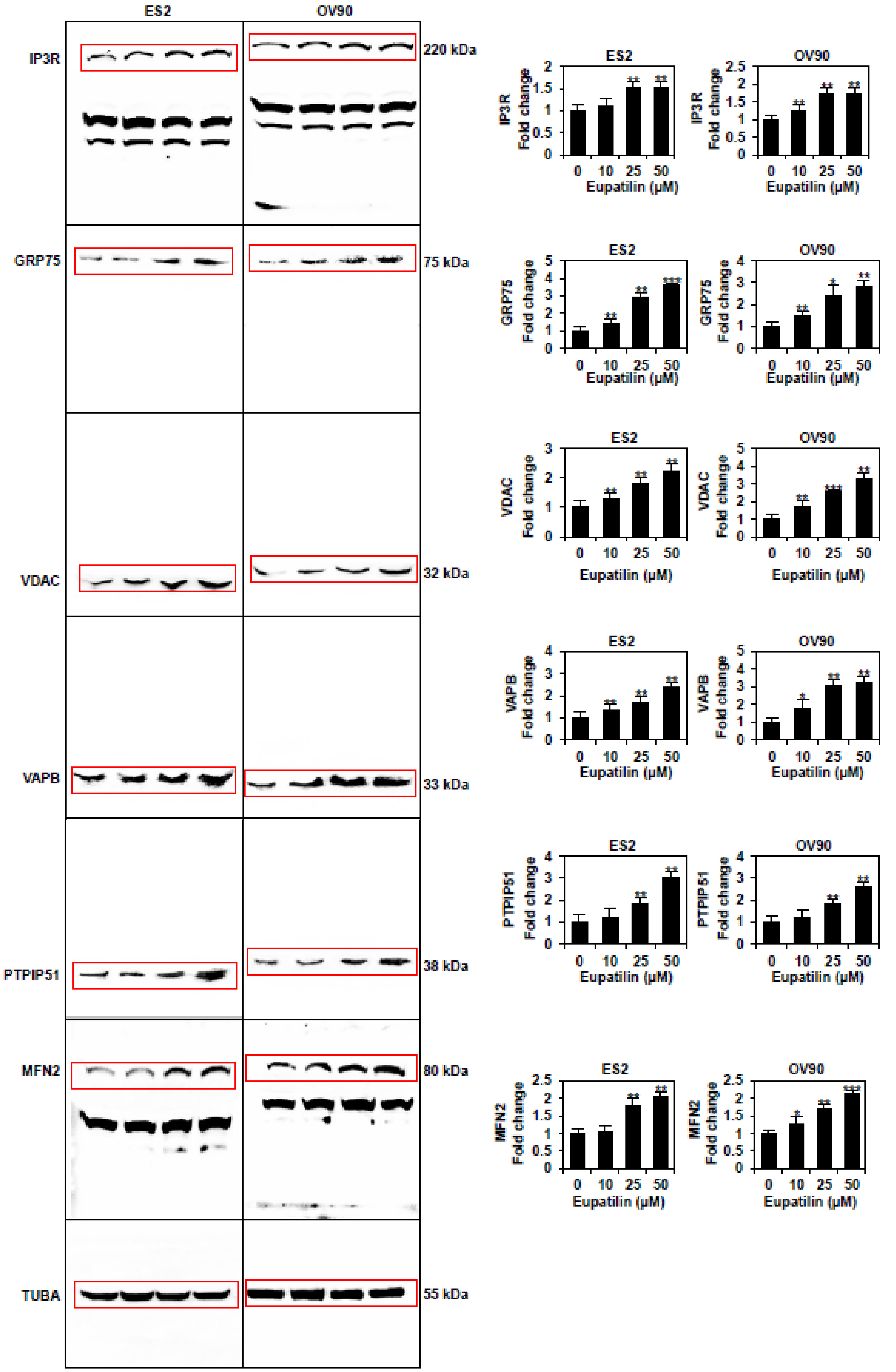

Figure S2. Detailed information about western blot in Figure 3.

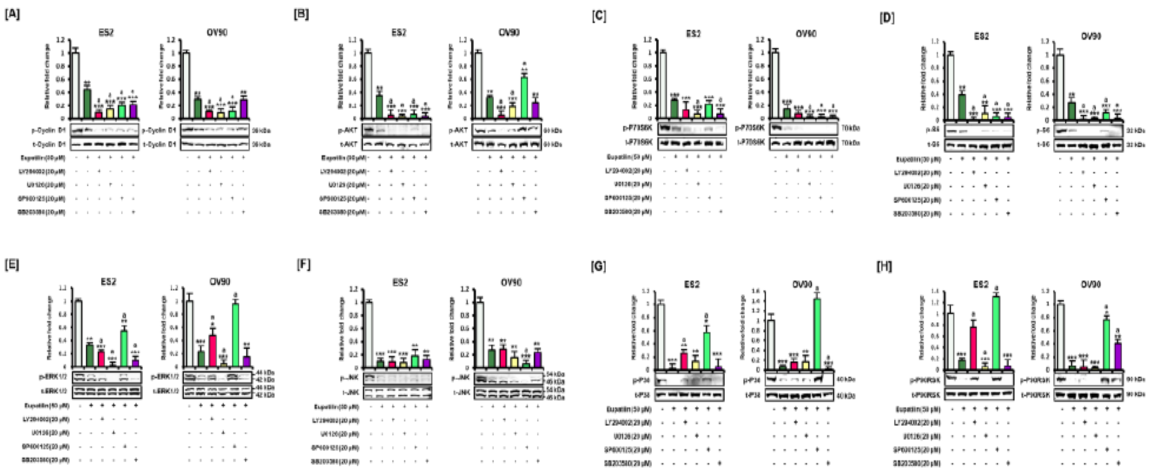

Figure S3. Dose-dependent effects of pharmacological inhibitors and eupatilin on PI3K/AKT and MAPK signaling in ovarian cancer cells. (A–H) Activities of phosphorylated CCND1 (p-CCND1) (A), p-AKT (B), p-P70S6K (C), p-S6 (D), p-ERK1/2 €, p-JNK (F), p-P38 (G), and p-P90RSK (H) protein levels were evaluated with each pharmacological inhibitor in eupatilin-treated ovarian cancer cells. Each immunoblot calculated using the normalized ratio of phosphorylated protein relative to the total protein. The experiments were performed in triplicate. Data represent the mean ± standard deviation, and asterisks indicate that the effect of treatment was statistically significant (* $p < 0.05$, ** $p < 0.01$, and *** $p < 0.001$). A lowercase letter (a) indicates significant changes ($p < 0.05$) compared with eupatilin alone. Detailed information about western blot can be found at Figure S4.

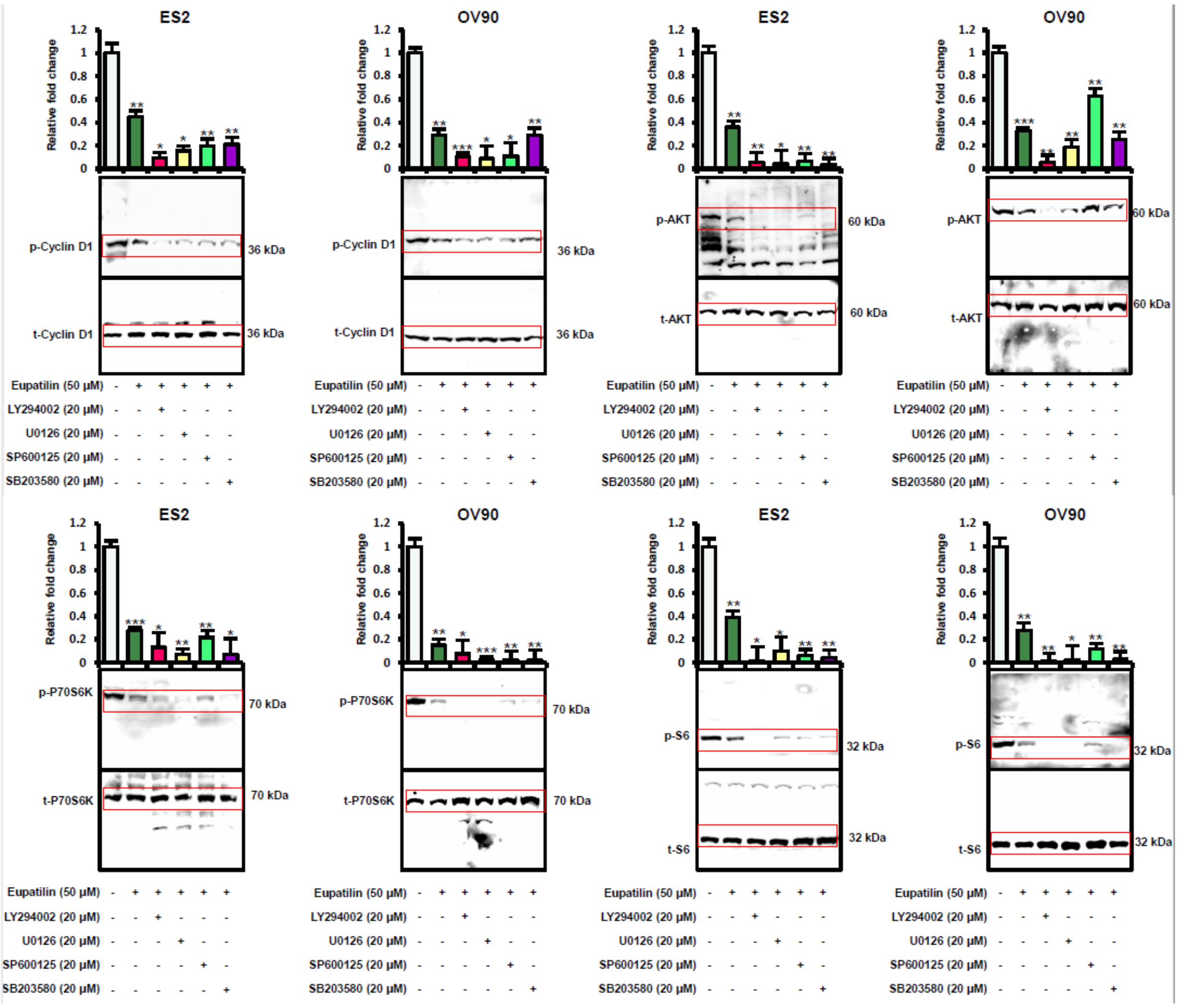

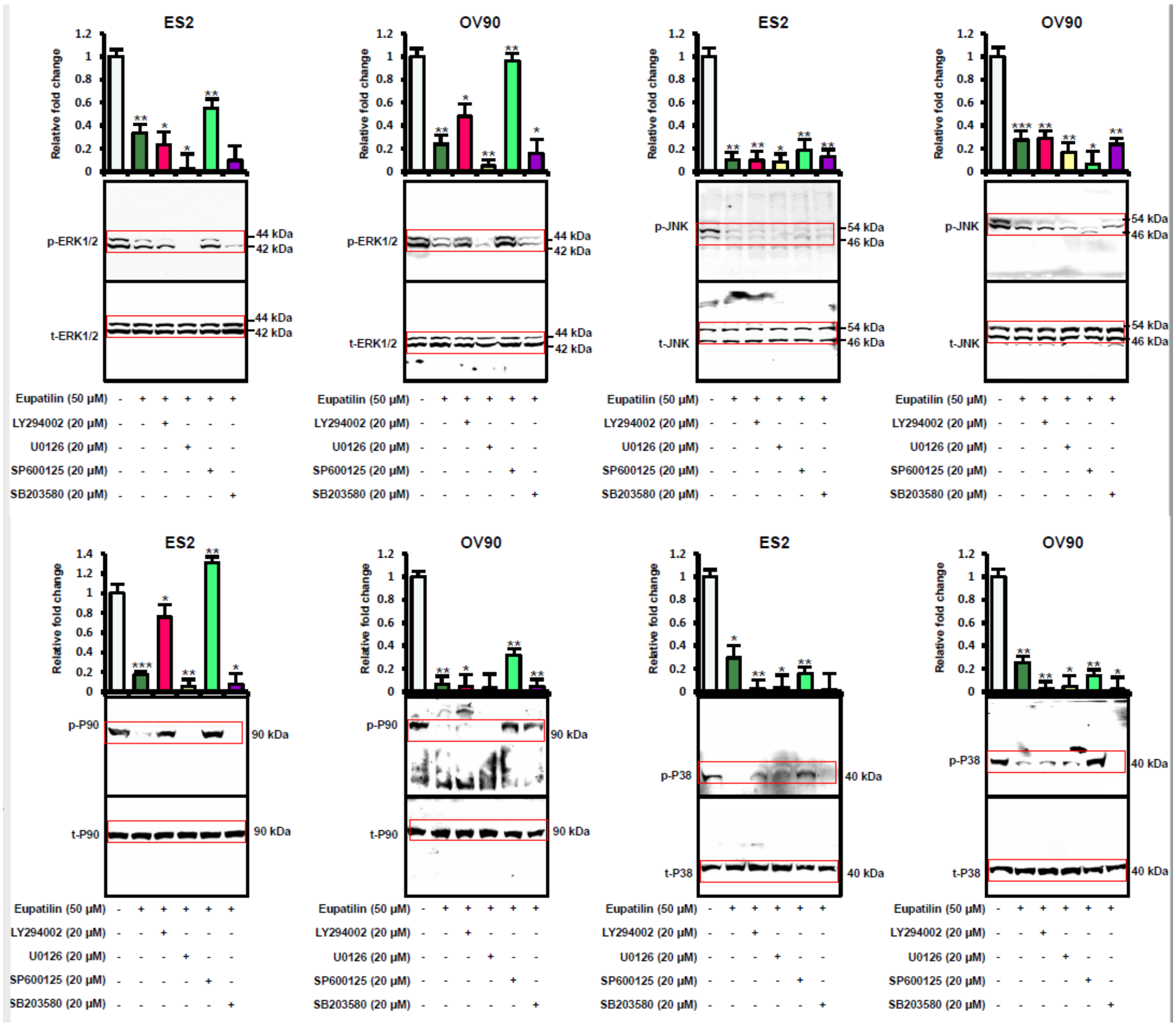

Figure S4. Detailed information about western blot in Figure S3.

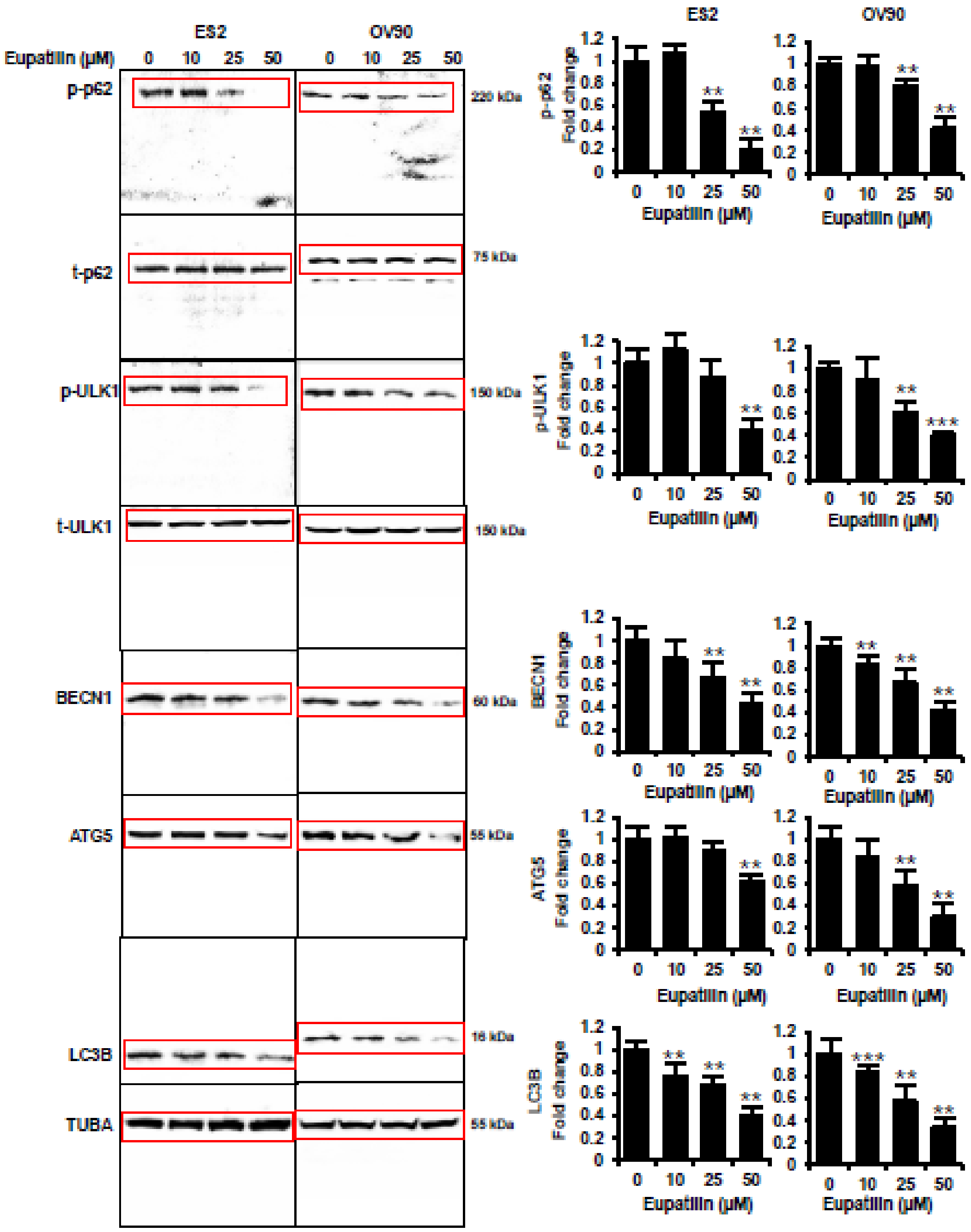
ES2
OV90
Eupatilin (μM)
0 10 25 50
p-p62
t-p62
p-ULK1
t-ULK1
BECN1
ATG5
LC3B
TUBA
220 kDa
75 kDa
150 kDa
60 kDa
55 kDa
16 kDa
p-p62 Fold change
p-ULK1 Fold change
BECN1 Fold change
ATG5 Fold change
LC3B Fold change
0 0.2 0.4 0.6 0.8 1 1.2
**
***

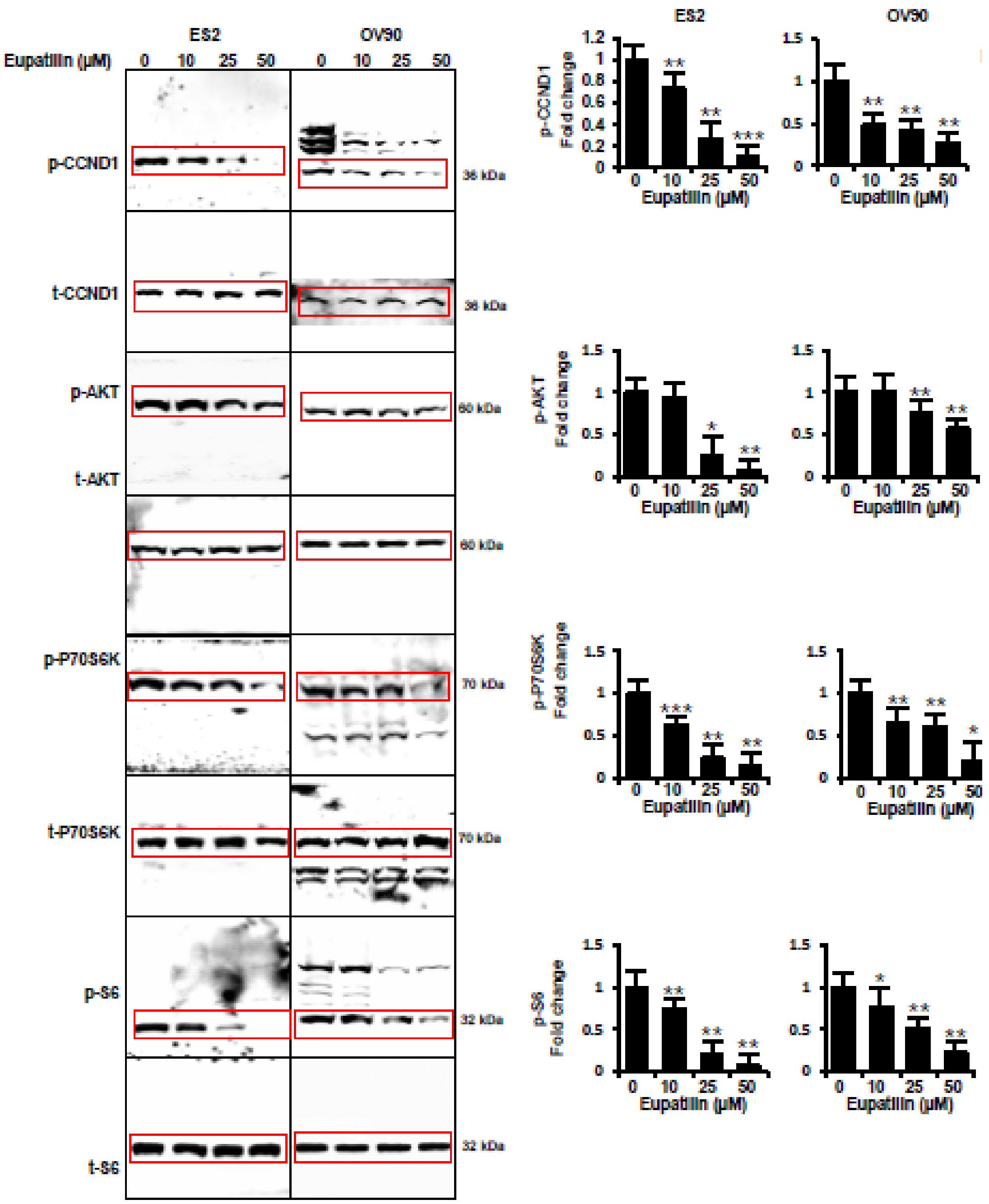
ES2
OV90
Eupatilin (μM)
0 10 25 50 0 10 25 50
p-CCND1
t-CCND1
p-AKT
t-AKT
p-P70S6K
t-P70S6K
p-S6
t-S6
36 kDa
60 kDa
70 kDa
32 kDa
p-CCND1 Fold change
p-AKT Fold change
p-P70S6K Fold change
p-S6 Fold change
Eupatilin (μM)

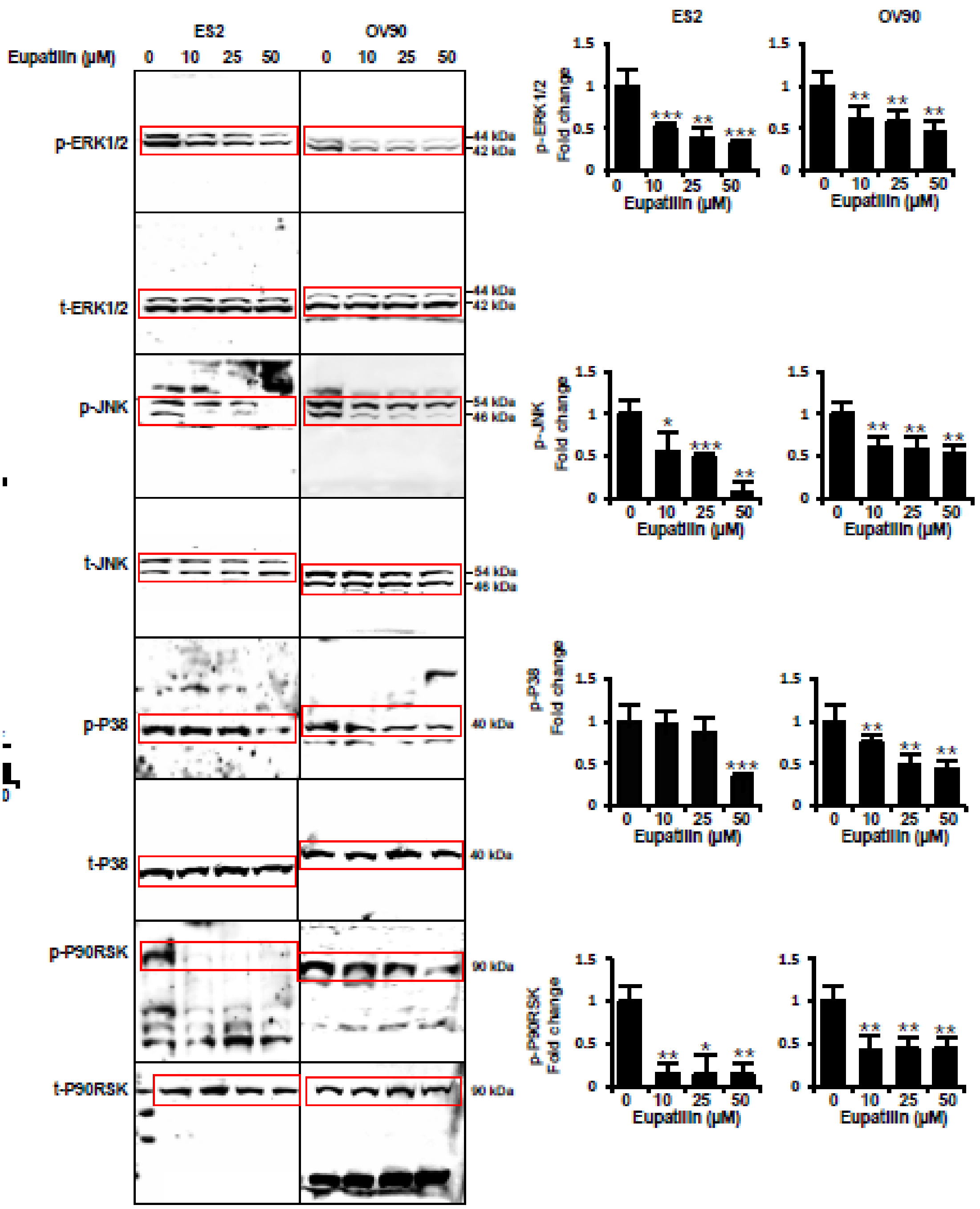

Figure S5. Detailed information about western blot in Figure 4.

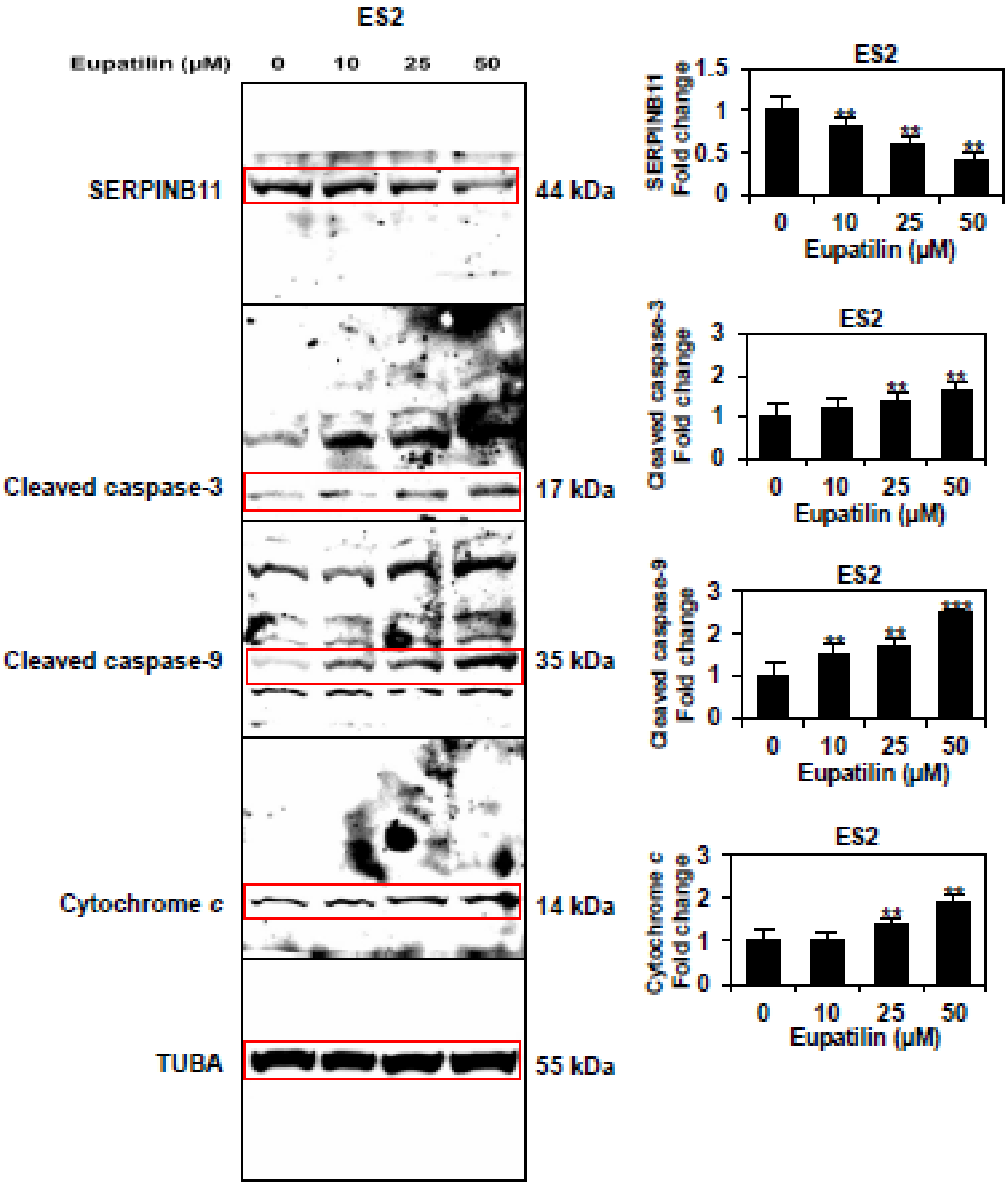
ES2
Eupatilin (μM) 0 10 25 50
SERPINB11 44 kDa
Cleaved caspase-3 17 kDa
Cleaved caspase-9 35 kDa
Cytochrome c 14 kDa
TUBA 55 kDa
ES2
SERPINB11 Fold change
Cleaved caspase-3 Fold change
Cleaved caspase-9 Fold change
Cytochrome c Fold change
Eupatilin (μM)

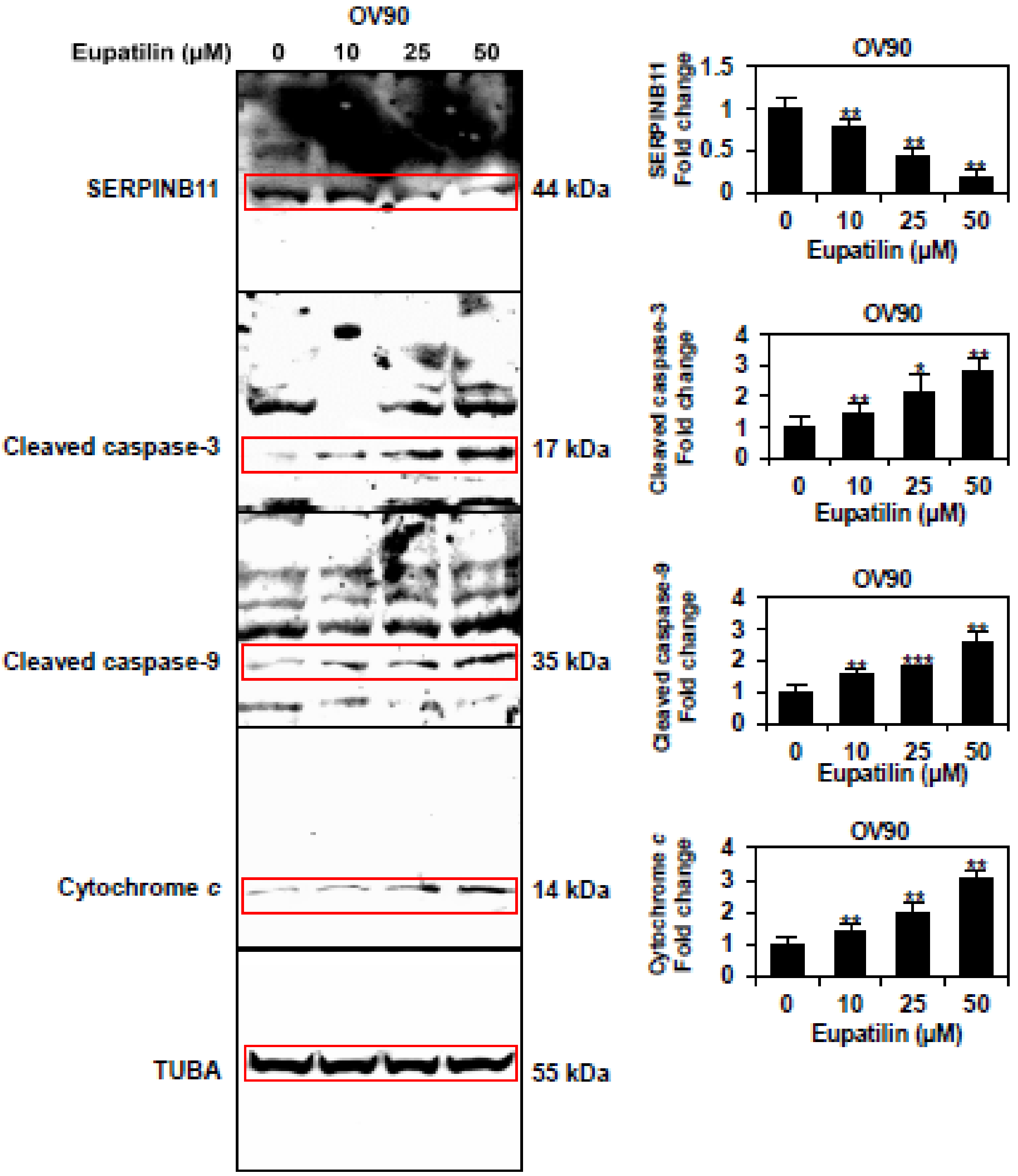
OV90
Eupatilin (μM) 0 10 25 50
SERPINB11
44 kDa
Cleaved caspase-3
17 kDa
Cleaved caspase-9
35 kDa
Cytochrome c
14 kDa
TUBA
55 kDa
OV90
SERPINB11 Fold change
Cleaved caspase-3 Fold change
Cleaved caspase-9 Fold change
Cytochrome c Fold change
Eupatilin (μM)
0 10 25 50

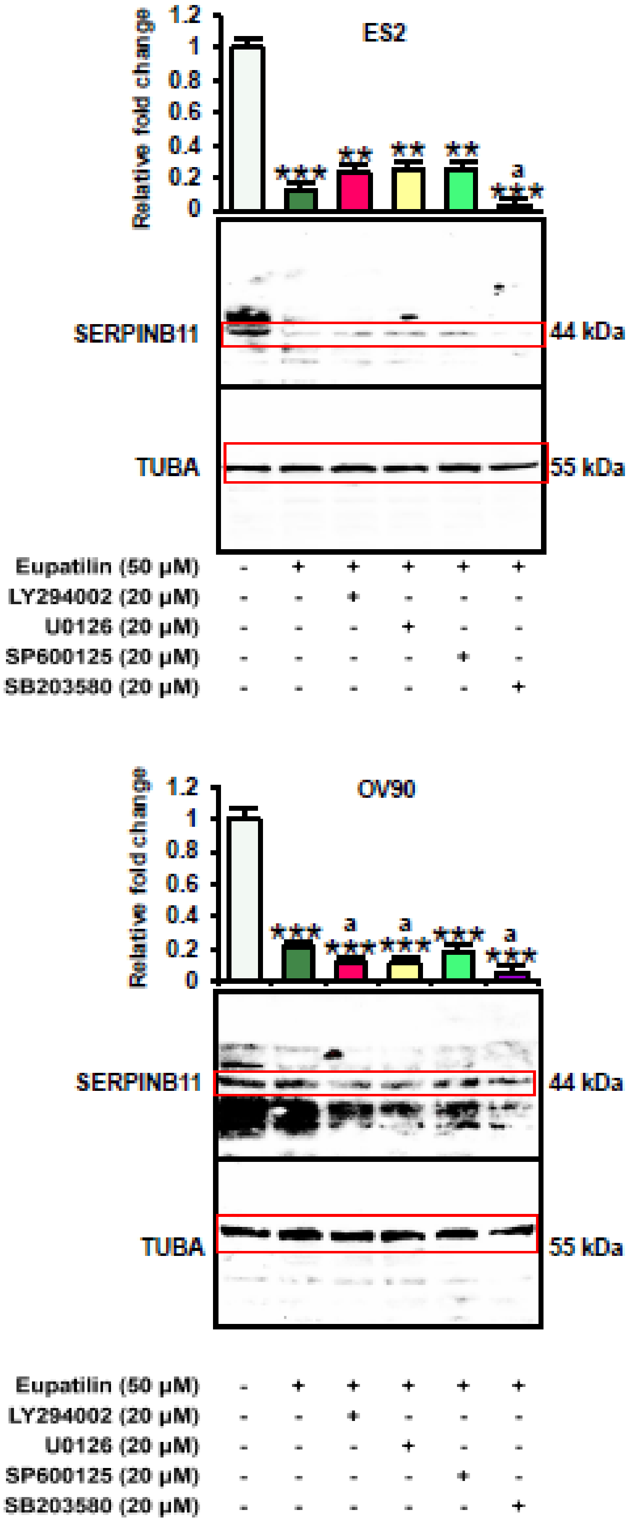

Figure S6. Detailed information about western blot in Figure 5.

© 2020 by the authors. Licensee MDPI, Basel, Switzerland. This article is an open access article distributed under the terms and conditions of the Creative Commons Attribution (CC BY) license (http://creativecommons.org/licenses/by/4.0/).
